# Supplementary figures and images for: Identification of common predisposing loci to hematopoietic cancers in four dog breeds
Source: PLoS Genet. 2021 Apr 1;17(4):e1009395. doi: 10.1371/journal.pgen.1009395 (PMC8016107; doi:10.1371/journal.pgen.1009395)

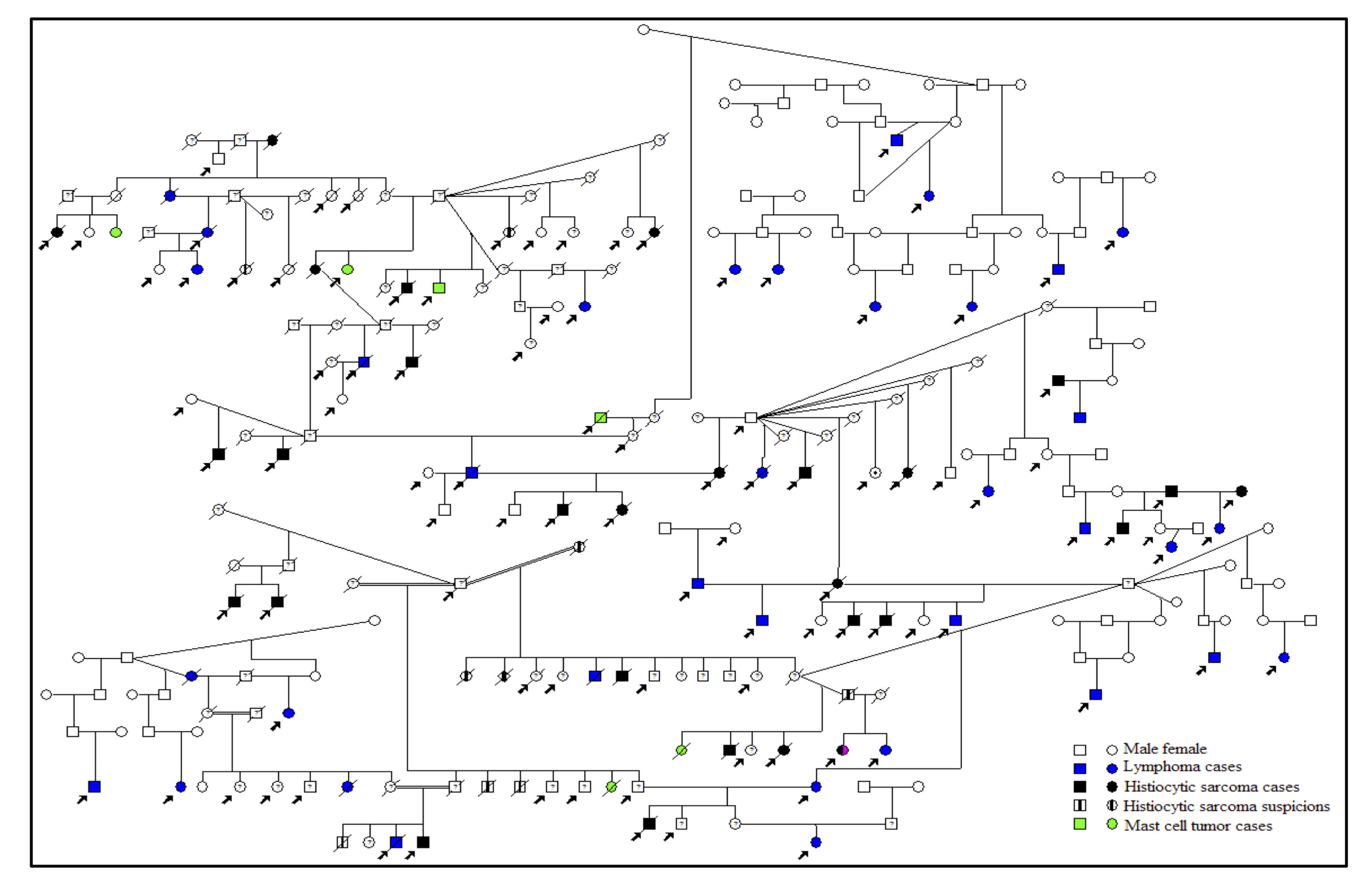

Supplement: S1 Fig — Pedigrees of a Bernese mountain dog family showing the co-segregation of lymphoma (blue) and mast cell tumor (green) with histiocytic sarcoma (black). (TIFF) [file pgen.1009395.s001.tiff]

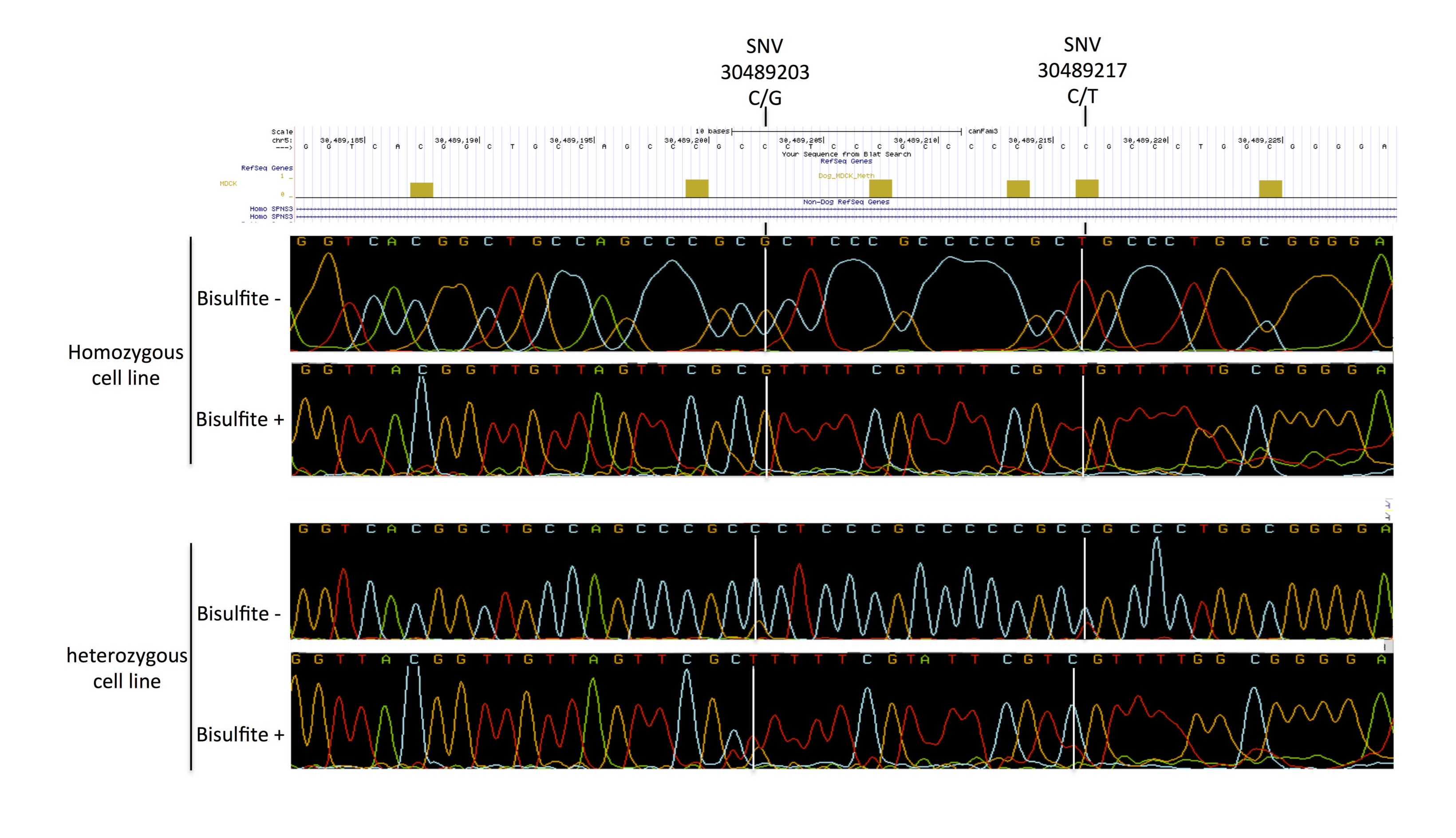

Supplement: S4 Fig — The UCSC track of chr5:30,489,183–30,489,230 with methylation track (Dog-MDCK-Meth) and Sanger sequencing performed on histiocytic sarcoma cell lines are represented. Sequencing of homozygous and heterozygous histiocytic sarcoma cell lines in the absence and presence of bisulfite treatment showed that the two SNVs present with allele-specific methylation in histiocytic cells. (TIFF) [file pgen.1009395.s004.tiff]

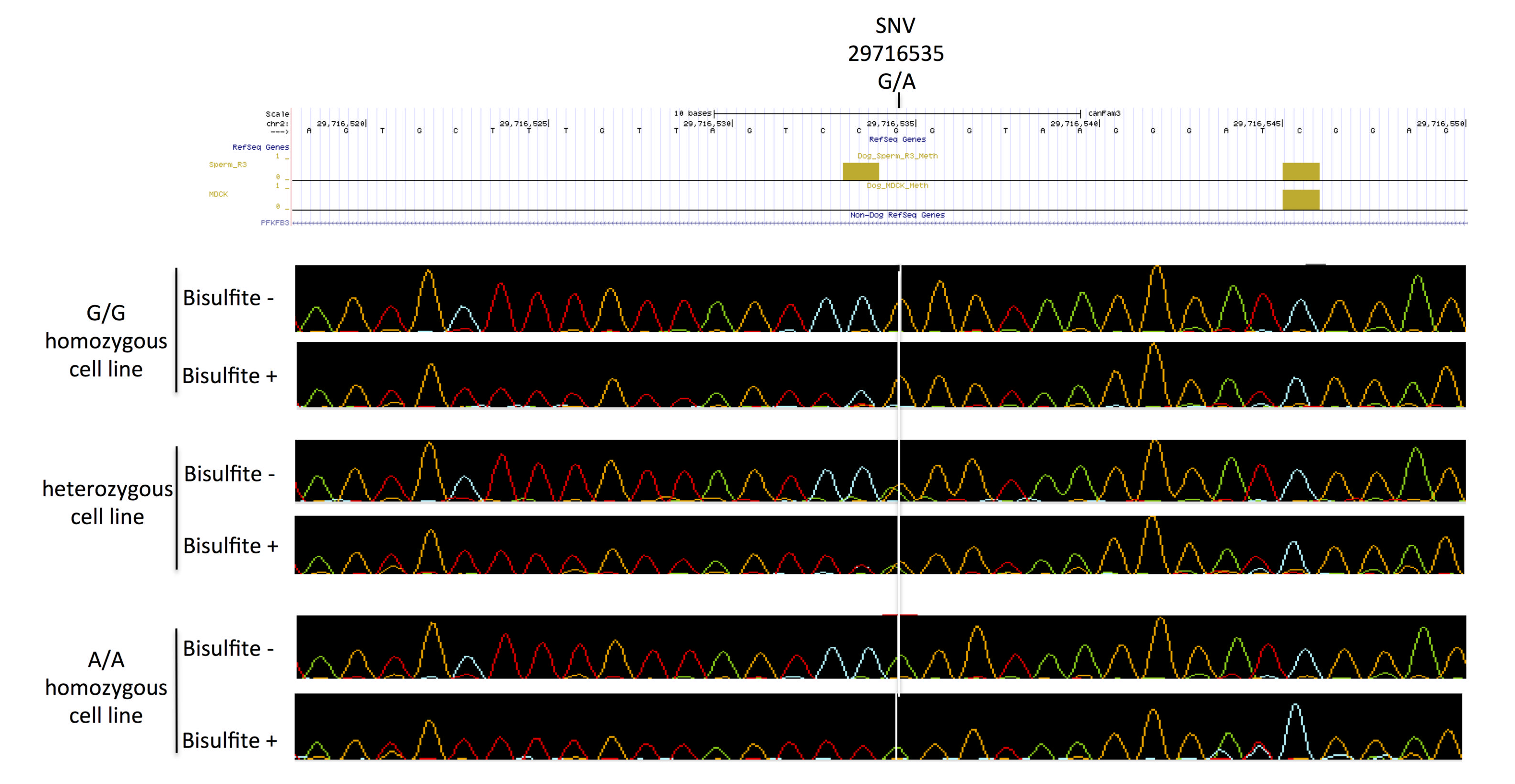

Supplement: S5 Fig — The UCSC track of chr2:29,716,519–29,716,550 with methylation tracks (Dog-MDCK-Meth, Dog-R3-Sperm-Meth) and Sanger sequencing performed on histiocytic sarcoma cell lines are represented. Sequencing of homozygous and heterozygous histiocytic sarcoma cell lines in the absence and presence of bisulfite treatment showed that the SNV presents with allele-specific methylation in histiocytic cell lines. (TIFF) [file pgen.1009395.s005.tiff]

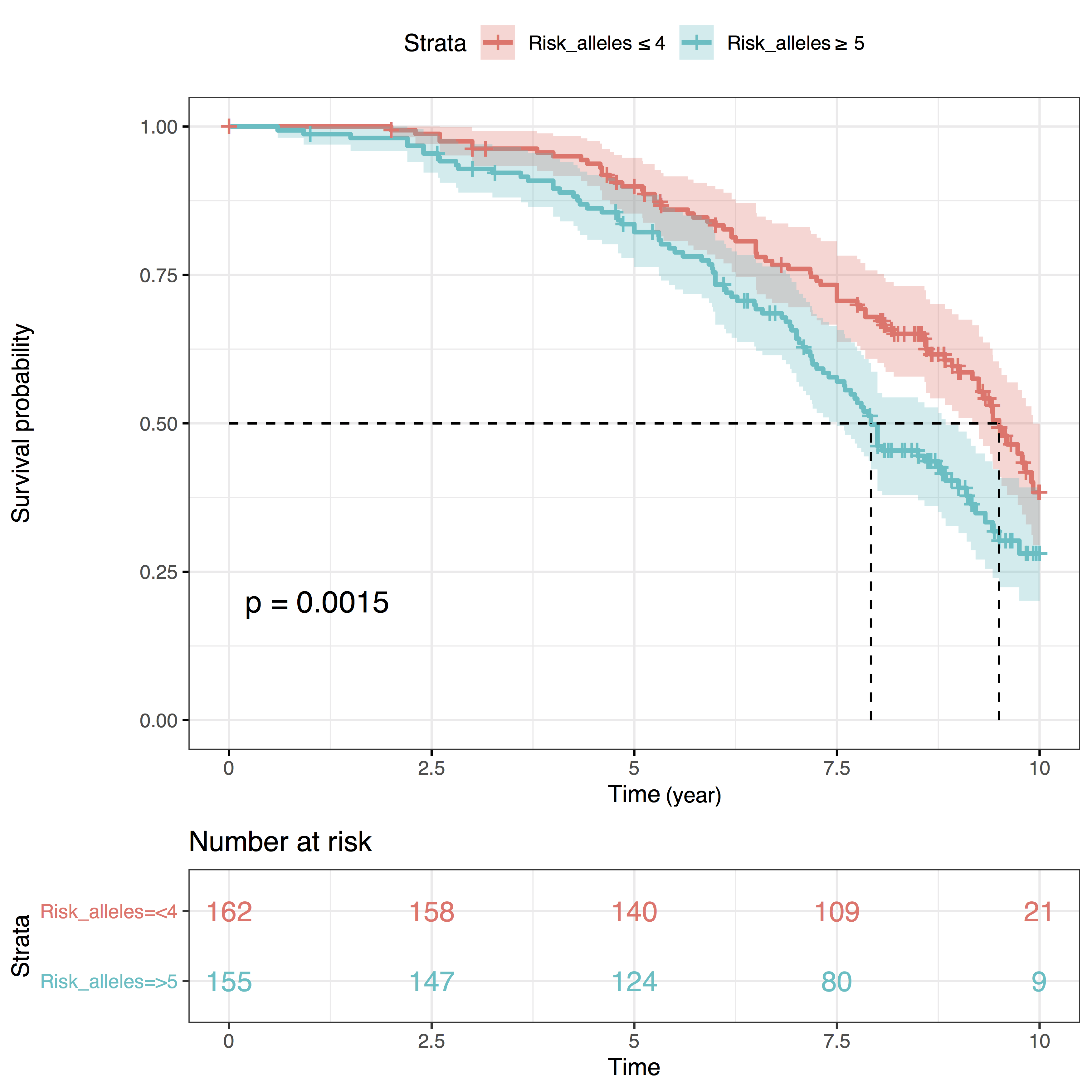

Supplement: S6 Fig — Kaplan–Meier estimates of BMD longevity and the corresponding hazard ratio is represented according to the number of risk alleles (n ≤ 4 or n ≥ 5). Survival mean and survival median are 8.4 and 9.5 years, respectively, for BMDs with ≤ 4 risk alleles; whereas, the survival mean and survival median are 7.54 and 7.92 years, respectively, for BMDs with ≥5 risk alleles. (TIFF) [file pgen.1009395.s006.tiff]

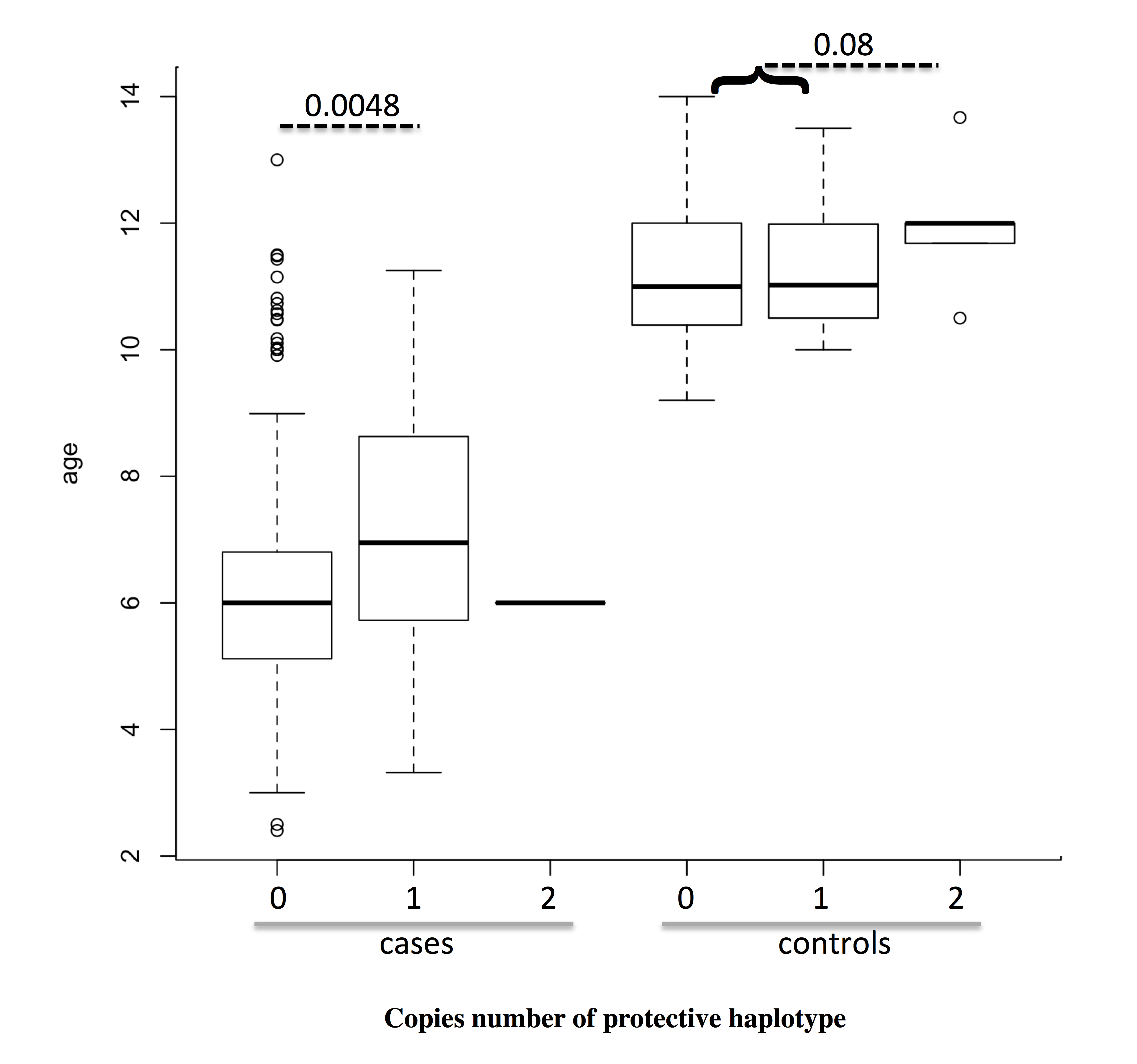

Supplement: S7 Fig — Cases with zero copies, n = 183, mean age = 6.22 years. Cases with one copy, n = 24, mean age = 7.23 years. Cases with two copies, n = 1, mean age = 6.9 years. Controls with zero copies, n = 132, mean age = 11.21 years. Controls with one copy, n = 50, mean age = 11.27 years. Controls with two copies, n = 5, mean age = 11.97 years. One-sided Wilcoxon rank sum test was conducted. (TIFF) [file pgen.1009395.s007.tiff]
