# Supplementary material for: Identification of common predisposing loci to hematopoietic cancers in four dog breeds
Source: PLoS Genet. 2021 Apr 1;17(4):e1009395. doi: 10.1371/journal.pgen.1009395 (PMC8016107; doi:10.1371/journal.pgen.1009395)
Supplement: Attachment — Submitted filename: nihms-437755.pdf [file pgen.1009395.s014.pdf]

NIH Public Access
Author Manuscript
Hum Genet. Author manuscript; available in PMC 2014 May 01.

Published in final edited form as:
Hum Genet. 2013 May ; 132(5): 509–522. doi:10.1007/s00439-013-1266-7.

Imputation across genotyping arrays for genome-wide association studies: assessment of bias and a correction strategy

Eric O. Johnson,
Behavioral Health Epidemiology Program, RTI International, 3040 Cornwallis Road, PO Box 12194, Research Triangle Park, NC 27709-12194, USA

Dana B. Hancock,
Behavioral Health Epidemiology Program, RTI International, 3040 Cornwallis Road, PO Box 12194, Research Triangle Park, NC 27709-12194, USA

Joshua L. Levy,
Research Computing Division, RTI International, Research Triangle Park, NC 27709, USA

Nathan C. Gaddis,
Research Computing Division, RTI International, Research Triangle Park, NC 27709, USA

Nancy L. Saccone,
Department of Genetics, Washington University, St. Louis, MO 63110, USA

Laura J. Bierut, and
Department of Psychiatry, Washington University, St. Louis, MO 63110, USA

Grier P. Page
Genomics, Statistical Genetics, and Environmental Research Program, RTI International, Atlanta, GA 30341, USA

Abstract

A great promise of publicly sharing genome-wide association data is the potential to create composite sets of controls. However, studies often use different genotyping arrays, and imputation to a common set of SNPs has shown substantial bias: a problem which has no broadly applicable solution. Based on the idea that using differing genotyped SNP sets as inputs creates differential imputation errors and thus bias in the composite set of controls, we examined the degree to which each of the following occurs: (1) imputation based on the union of genotyped SNPs (i.e., SNPs available on one or more arrays) results in bias, as evidenced by spurious associations (type 1 error) between imputed genotypes and arbitrarily assigned case/control status; (2) imputation based on the intersection of geno-typed SNPs (i.e., SNPs available on all arrays) does not evidence

© Springer-Verlag Berlin Heidelberg 2013

Correspondence to: Eric O. Johnson.
ejohnson@rti.org.

Electronic supplementary material The online version of this article (doi:10.1007/s00439-013-1266-7) contains supplementary material, which is available to authorized users.

Web resources
dbGaP, http://www.ncbi.nlm.nih.gov/projects/gap/
EGA, https://www.ebi.ac.uk/ega/
EIGENSTRAT, http://genepath.med.harvard.edu/~reich/Software.htm
KING, http://people.virginia.edu/~wc9c/KING/Download.htm
PLINK, http://pngu.mgh.harvard.edu/~purcell/plink/

NIH-PA Author Manuscript
NIH-PA Author Manuscript
NIH-PA Author Manuscript

NIH-PA Author Manuscript

NIH-PA Author Manuscript

NIH-PA Author Manuscript

such bias; and (3) imputation quality varies by the size of the intersection of genotyped SNP sets. Imputations were conducted in European Americans and African Americans with reference to HapMap phase II and III data. Imputation based on the union of genotyped SNPs across the Illumina 1M and 550v3 arrays showed spurious associations for 0.2 % of SNPs: ~2,000 false positives per million SNPs imputed. Biases remained problematic for very similar arrays (550v1 vs. 550v3) and were substantial for dissimilar arrays (Illumina 1M vs. Affymetrix 6.0). In all instances, imputing based on the intersection of genotyped SNPs (as few as 30 % of the total SNPs genotyped) eliminated such bias while still achieving good imputation quality.

Introduction

Centralized repositories for genome-wide association study (GWAS) data, such as the database of Genotypes and Phenotypes (dbGaP) and the European Genome-phenome Archive (EGA), were established to encourage data sharing in an effort to advance medical science while maximizing use of publicly funded resources. One of the great promises of publicly sharing GWAS data through these repositories is the potential to create composite sets of public controls for new studies. Combining phenotypic and genotypic data from several studies into a single population control group and pairing these combined data with cases of the phenotype of interest allow for powerful opportunities to identify new genetic associations. Publicly available controls might also be used to augment study controls to increase sample size and boost statistical power (Ho and Lange 2010; Mukherjee et al. 2011; Zhuang et al. 2010). These study designs provide a cost-effective strategy to obtain the large number of control subjects needed for GWAS analyses, which may be particularly beneficial for ancestry groups with fewer available samples (e.g., African Americans) (Hartz et al. 2011). However, publicly available GWAS differ in many respects, including use of a wide variety of high-density genotyping arrays. Thus, one of the substantial challenges such studies face is creating a common set of single nucleotide polymorphisms (SNPs) across studies contributing to composite controls and study cases.

Statistical imputation of untyped SNP genotypes based on reference haplotype panels can be used to overcome this challenge. Imputation has been primarily applied to increase the SNP density for analysis in studies where cases and controls were recruited together and genotyped in a uniform fashion on the same array at the same time, reducing the risk of batch effects that impact SNP genotype calling. In the case of composite public controls derived from multiple studies genotyped on different arrays, variations in genotyping protocols create systematic differences, which introduce the potential for differential error in estimated allele probabilities at each of the imputed markers and artifactual differences in allele frequencies. These artifacts might manifest in significant statistical bias in downstream tests of genotype–phenotype association.

Sinnott and Kraft (2012) and Uh et al. (2012) recently have demonstrated that substantial false positive rates occur when imputation is used to create a common set of SNPs for cases and controls genotyped on different arrays (Affymetrix vs. Illumina), which is analogous to combining controls from multiple studies as investigated in this study. Attempts to address this bias by adjusting for array effects using principal components failed (Sinnott and Kraft 2012). Post-imputation filtering of imputed SNPs required extreme thresholds on quality measures (R^2 and $R_T^2 \geq 0.98$), which did not fully remove false positive associations and left only 30 % of SNPs for analysis, substantially reducing statistical power for subsequent analyses (Sinnott and Kraft 2012; Uh et al. 2012). Thus, if the promise of using composite controls is to be realized on a large scale, alternative approaches of stringently limiting imputation-induced bias need to be developed.

NIH-PA Author Manuscript

NIH-PA Author Manuscript

NIH-PA Author Manuscript

In this study, we used data from GWAS repositories to estimate the magnitude of imputation-induced bias in a common set of SNPs among European Americans and African Americans genotyped on different Illumina and Affymetrix arrays. We hypothesize that using differing sets of genotyped SNPs from the different arrays as inputs creates differential imputation accuracy across samples resulting in the bias and spurious associations observed by others (Sinnott and Kraft 2012; Uh et al. 2012). However, in contrast to these studies which imputed each sample separately based on their differing sets of genotyped SNPs and then combined the imputed data for analysis (Sinnott and Kraft 2012; Uh et al. 2012) or used imputed data for some samples and genotyped data for other samples (Uh et al. 2012), we tested an intersection strategy in which we selected only the SNPs genotyped on all arrays for the samples to be combined and then imputed up to a common set of HapMap SNPs for analyses from a common set of genotyped SNPs. To test this hypothesis and correction strategy, we examined the degree to which each of the following occurs: (1) imputation across arrays based on the union of genotyped SNPs (i.e., SNPs available on one or more arrays) results in bias as evidenced by spurious associations (type 1 error) between imputed genotypes and arbitrarily assigned case/control status; (2) imputation across arrays based on the intersection of SNPs genotyped on all arrays does not evidence such bias; and (3) imputation quality varies by the size of the overlap of the intersection of genotyped SNPs across arrays. Finally, we examined the conditions under which using public controls adds sufficiently to a study's power that the additional study complexity and administrative work to obtain public controls is worth the effort, considering the balance of sample size and imputation accuracy.

Subjects and methods

Study subjects and genotyping arrays

Table 1 lists the sources of European American and African American study subjects, who were genotyped on one of three Illumina arrays (Human1M, HumanHap550 version 1, or HumanHap550 version 3) or the Affymetrix 6.0 array. All genotype data from European American study subjects were obtained from dbGaP. The availability of African American studies in dbGaP is more limited, so we utilized both dbGaP and Illumina's iControl database (Illumina, Inc., San Diego, CA, USA) to obtain genotype data on African American control subjects. All subject data were anonymous and publically available based on which the RTI International Institutional Review Board granted a human subjects exemption for this study.

Quality control

Quality control (QC) procedures, mimicking standard procedures used for GWAS, were conducted in each study separately using PLINK (Purcell et al. 2007) unless otherwise stated. Subjects were excluded due to call rate <95 %, discordance between reported gender and estimated gender based on chromosome X SNP data (F_{ST} < 0.2 used to indicate female and F_{ST} >0.8 used to indicate male), or excessive homozygosity based on autosomal SNP data (F_{ST} < −0.2 or F_{ST} >0.5). Further, for subject pairs having identity-by-state estimates greater than 99 % (indicative of sample duplication or monozygotic twins), we retained the subject with the highest call rate. Identity-by-descent (IBD) estimates were also generated to identify subject pairs (or clusters) with cryptic relatedness. For subjects classified as European American, we identified relative clusters having IBD >10 % (indicative of third-degree relation or closer) and retained the single subject having the highest call rate from each cluster. Since IBD estimates may be inflated in the presence of population stratification, we used the KING program (Manichaikul et al. 2010) to identify clusters among African American subjects. The KING program was designed specifically to circumvent the inflation of IBD estimates due to population stratification (Manichaikul et al.

2010). We used the IBD threshold of 10 % and the KING kinship coefficient threshold and retained the single subject having the highest call rate from each African American relative cluster.

Subjects were further evaluated for population structure to identify ancestral outliers using HapMap populations of European Americans (denoted CEU), Africans (denoted YRI), and Chinese (denoted CHB) for comparison in the STRUCTURE program (Pritchard et al. 2000). African American subjects having <60 % African ancestry were excluded. No European Americans were excluded due to ancestral misclassification.

Additional subject exclusions were made in dbGaP studies to remove the original study cases [e.g., alcohol dependent cases from the Study of Addiction: Genetics and Environment (SAGE) (Bierut et al. 2010)] (Table 1). No phenotypic exclusions were made for the iControl subjects, since no phenotype information is provided in the iControl database. Following all subject-level QC, genotyped SNPs were excluded due to minor allele frequency (MAF) <1 %, call rate <95 %, or Hardy–Weinberg equilibrium $P < 0.0001$. Numbers of genotyped subjects and polymorphic SNPs before and after QC are provided in Table 1.

Combining subjects genotyped on Illumina versus Affymetrix arrays required an additional QC step to remove SNPs with indeterminate or flipped strand orientation. Specifically, we removed SNPs with ambiguous alleles (i.e., SNPs with A/T or G/C alleles), due to problems with determining strand orientation between the Illumina versus Affymetrix arrays. Then, we used the flip option in PLINK to recode SNPs with an opposing strand orientation relative to the HapMap reference panel. After flipping, we removed a small number of SNPs with misassignment of allele code based on discrepant allele frequencies between the two arrays. The remaining SNPs were used as the input genotypes for imputation.

Reference haplotype panels

For genotype imputation in European Americans, we used the CEU reference haplotype panel from merged HapMap phase II + III data. For African Americans, we created a reference haplotype panel by combining HapMap phase II and III data from YRI, CEU, and ASW subjects. We previously found this specific reference panel to achieve optimal imputation quality and accuracy, compared to other combined panels from HapMap (unpublished data).

Imputation procedure

SNP imputation procedures use haplotype information on genotyped SNPs in the study population and predict untyped SNPs based on linkage disequilibrium (LD) patterns between SNPs, as estimated from reference panels of much denser genotyping, usually from HapMap (Altshuler et al. 2010) and more recently the 1000 Genomes project (Durbin et al. 2010). See Marchini and Howie for a review of this literature (Marchini and Howie 2010).

Genotype imputations reported here were conducted using MaCH, unless otherwise stated (Li et al. 2010). As other studies have done, (Shriner et al. 2010; Southam et al. 2011; Howie et al. 2011) we focused on a single chromosome (chromosome 22) for efficiently evaluating imputation performance. Imputation across genotyping arrays may be conducted separately within each originating study or with all study subjects combined. Here, we report results from separate imputation in each of the eight originating studies of European Americans or African Americans (Table 1). Similar results were found from imputations conducted using all study subjects combined (results not shown).

NIH-PA Author Manuscript NIH-PA Author Manuscript NIH-PA Author Manuscript

NIH-PA Author Manuscript

NIH-PA Author Manuscript

NIH-PA Author Manuscript

The first imputation step in MaCH used a subset of 200 randomly selected haplotypes from study subjects to estimate model parameters (crossover and error rates). Genotype imputation was then conducted in the full study populations using the model parameter estimates from the previous round. R^2 values in the MaCH output (which are the estimated squared correlation between each imputed genotype and its true underlying genotype) were used to assess imputation quality.

Statistical analyses

Imputation results were compared across subjects genotyped on different arrays by arbitrarily assigning subjects from one originating study as cases and subjects from the other originating study as controls. Associations between SNP genotype dosage (fractional value ranging from 0 to 2.0 that corresponds to the estimated reference allele count) and the assigned case-control status were tested using a logistic regression model implemented in PLINK (Purcell et al. 2007). To eliminate any potential bias from residual population stratification, we applied EIGENSTRAT (Price et al. 2006) analysis to each set of study comparisons using a set of autosomal SNPs, which included only those having $R^2 < 0.2$ within a 1,500 window size and omitted known regions of high LD, as implemented elsewhere (Fellay et al. 2007). The first ten principal components were included as covariates in all regression models.

Three data sets were compared for each pair of studies: (1) genotyped SNPs shared on both arrays; (2) imputed SNPs based on the union of genotyped SNPs available on either array; and (3) imputed SNPs based on the intersection of genotyped SNPs available on both arrays. The first analysis tested for any potential genotyping bias that might affect imputation results. The second and third analyses were designed to test the magnitude of bias resulting from imputing the same SNPs based on either the union of genotypes SNPs (which uses the maximal information available) or the intersection of genotyped SNPs across arrays (which corresponds to less input information). Statistically significant SNP associations were identified as those having $P < 1 \times 10^{-6}$, based on Bonferroni correction for the largest number of SNPs in any one of our analyses ($N = 43{,}035$ SNPs). Since case or control status was arbitrarily assigned, inflated λ_{gc} values and significant associations between SNPs and case status demonstrate systematic imputation bias as evidenced by false positive or spurious associations.

Calculating statistical power for using public controls under cross array imputation scenarios

Adding publically available controls to augment existing study controls or using such public controls in lieu of study controls would be an attractive option to substantially increase sample size and power in the absence of cross array imputation-induced bias (Ho and Lange 2010). However, imputation engenders error in the estimated allele count across imputed SNPs, indicated by average R^2, which reduces effective sample size (Pritchard and Przeworski 2001; Pasaniuc et al. 2012). We compared the effects of increasing sample size and potentially poorer imputation accuracy as the number of samples genotyped on different arrays increases under two scenarios: (1) adding public controls to a fixed sample of 2,000 study cases and 2,000 study controls; and (2) focusing on the study design stage where we have fixed resources to ascertain and genotype 4,000 individuals with differing mixes of study cases, study controls, and public controls. Under both scenarios, we began with a baseline model in which a study has 2,000 cases and 2,000 controls genotyped, providing 80 % power to detect an additive SNP effect size of 1 % variance explained in the phenotype at genome-wide significance ($P \leq 5 \times 10^{-8}$). This is equivalent to detecting a minimum odds ratio of 1.545, 1.405, 1.355, and 1.335 for SNPs with MAFs of 10, 20, 30, and 40 %, respectively, with the same sample size. The calculations were made following Zheng et al.

NIH-PA Author Manuscript

NIH-PA Author Manuscript

NIH-PA Author Manuscript

(2011) simulation of power by imputation accuracy (average R^2) for a standard 1 degree of freedom test under an additive genetic model for imputed allele dosage. Modification to sample size and proportion of controls to cases were made taking the harmonic mean of the numbers of cases and controls multiplied by two to produce the overall sample size for a given scenario. We applied a given level of average R^2 to proportionately reduce sample size to effective sample size (Pritchard and Przeworski 2001; Pasaniuc et al. 2012) due to imputation inaccuracy, and then used Elston's Excellent Estimator (Tiwari et al. 2011) via the web tool Analytic Power Calculation (http://gwatestdriver.ssg.uab.edu/) to estimate power for a given sample scenario (at various α, β, and sample sizes) and level of imputation accuracy (average R^2).

Results

Imputation within the Illumina family of arrays

The first assessment of cross array-induced imputation bias combined study subjects genotyped on 1M from SAGE (Bierut et al. 2010) with subjects genotyped on 550v3 from the Cancer Genetic Markers of Susceptibility (CGEMS) Pancreatic Cancer Cohort Consortium (PanScan) (Amundadottir et al. 2009) for European Americans or iControl for African Americans. Figure 1 presents the λ_{gc} values and percentages of SNPs with false positive associations ($P < 1 \times 10^{-6}$) for each of the three assessments of bias (genotyped SNPs shared on both arrays, imputation based on the union of genotyped SNPs across arrays, and imputation based on the intersection of genotyped SNPs across arrays). Tests of association between arbitrarily assigned case status and the genotyped SNPs shared on both arrays showed no statistically significant associations (Fig. 1a, d), indicating that there was no genotyping bias between these arrays. However, association tests for SNPs imputed based on the union of genotyped SNPs available on either array revealed spurious results as indicated by inflated λ_{gc} values and SNPs having statistically significant P values across the MAF spectrum (Fig. 1b, e). Overall, 0.20 % of the imputed SNPs had $P < 1 \times 10^{-6}$ in both European Americans (71 false positives of 34,515 imputed SNPs) and African Americans (87 false positives of 42,963 imputed SNPs). This imputation strategy based on the union of genotyped SNPs incurred substantial deviation from expectation, as demonstrated in Figure S1. In contrast, conducting imputation based on the intersection of genotyped SNPs available on both arrays resulted in no spurious associations (Fig. 1c, f). To be sure that choice of software did not influence our observed pattern of results, we ran the Illumina 1M versus 550v3 comparisons in African Americans using IMPUTE2 and found parallel results to those obtained using MaCH (Figure S2).

Using the same Illumina 1M versus 550v3 comparisons, we evaluated whether the imputation-induced bias based on the union of genotyped SNPs differed according to high versus low LD patterns. We employed the LD pruning procedure in PLINK (Purcell et al. 2007) with a 1,500 SNP sliding window to select SNPs under high LD with other SNPs (squared correlation coefficient between SNPs [r^2] >0.8) and conversely SNPs in linkage equilibrium ($r^2 < 0.2$). SNPs were selected according to the LD patterns in the SAGE subjects, separately by ethnic group, and their SNP association results were taken from the comparison to PanScan for European Africans (Fig. 1b) or iControl for African Americans (Fig. 1d). In the European Americans, 0.64 % of the 2,984 imputed SNPs in low LD and 0.18 % of 23,072 imputed SNPs in high LD were false positives. In African Americans, 0.30 % of the 6,590 imputed SNPs in low LD and 0.18 % of 19,134 imputed SNPs in high LD were false positives.

Imputation-induced bias arises from the union of genotyped SNPs even across similar Illumina arrays (550v1 and 550v3). European American subjects from PanScan were compared to subjects from the CGEMS breast cancer GWAS (Hunter et al. 2007), and

African American subjects from two subsets of the iControl database were compared. No spurious SNP associations were found when testing genotyped SNPs available on both arrays or testing SNPs imputed based on the intersection of genotyped SNPs across both arrays (results not shown). Testing SNPs that were imputed based on the union of genotyped SNPs available on either array resulted in spurious SNP associations (Figure S3), albeit the percentages of SNPs showing bias were predictably smaller given the array similarities (0.07 % of imputed SNPs with $P < 1 \times 10^{-6}$ in each ancestry group).

Imputation across Illumina and Affymetrix arrays

To evaluate the bias induced by imputation across highly different arrays, we combined SAGE subjects genotyped on Illumina 1M with subjects genotyped on Affymetrix 6.0 from the Genetic Association Information Network (GAIN) GWAS of Schizophrenia (Manolio et al. 2007). European American and African American subjects from these studies were analyzed separately. A single false positive SNP association was observed among the genotype SNPs shared on both arrays in European Americans (Fig. 2a), but no false positive SNP associations were observed in African Americans (Fig. 2d). Compared to the analyses across the Illumina family of arrays, substantially more false positive associations were observed when using imputed SNPs based on the union of SNPs available on the Illumina or Affymetrix array (Fig. 2b, e): 184 (0.53 %) false positives of 34,503 imputed SNPs in European Americans and 271 (0.63 %) false positives of 43,035 imputed SNPs in African Americans. The deviations from expectation were substantial (Figure S4). Our strategy of using the intersection of SNPs as the basis for imputation was able to eliminate these biases, even when combining these highly different arrays (Fig. 2c, f).

Assessing the biased SNPs

Our investigation into the nature of the bias observed under the union-of-SNPs imputation strategy showed minimal overlap in the SNPs with spurious association between the two ancestry groups. Additionally, making post-imputation SNP exclusions for $R^2 < 0.3$ removed some, but not all of the spurious SNP associations (Figure S5). After the R^2 exclusion, the remaining SNPs with spurious association tended to have MAF <10 % (especially in European Americans) and/or large discrepancies in R^2 between the two studies (especially in African Americans).

Assessing the impact of the intersection strategy on SNP imputation quality

The unbiased intersection strategy for imputation across arrays uses fewer genotyped SNPs as the basis for imputation compared to the union strategy. We took two strategies to investigate the impact of using smaller numbers of SNPs in the intersection-based strategy on SNP imputation quality. First we evaluated the imputation quality of SNPs for which we had genotype data but were removed from the intersection set for imputation because they were not present in all arrays. This allowed us to compare the true genotypes to the genotypes imputed using the intersection strategy. In European Americans from SAGE, 40 % of the SNPs genotyped on the 1M array were not genotyped on the 550v3 and thus were masked for the intersection-based imputation strategy. For these SNPs, the “best call” imputed genotypes (Shriner et al. 2010) were highly concordant with their directly typed genotype (97.1 % concordance rate), and 99.6 % of the masked genotyped SNPs were imputed at $R^2 > 0.3$ (standard threshold for evaluating imputation quality, Li et al. 2010). A comparable analysis of the masked genotyped SNPs in SAGE African Americans resulted in a 94.7 % concordance rate and 97.1 % of the SNPs having $R^2 > 0.3$.

Second, we evaluated the effect of varying input genotyped SNP set sizes on overall imputation quality for the intersection-based strategy using the European American and African American control subjects from SAGE. Figure 3 compares the average R^2 by MAF

NIH-PA Author Manuscript NIH-PA Author Manuscript NIH-PA Author Manuscript

NIH-PA Author Manuscript

NIH-PA Author Manuscript

NIH-PA Author Manuscript

for imputed SNPs, resulting from a range of input genotyped SNP sets available from Illumina 1M and its intersections with one or more other arrays. The imputation procedures were iteratively repeated following the removal of 1M SNPs not available on various Illumina arrays (HumanOmni1-Quad, Human660W, 550v1, and HumanHap 300-Duo version 2) or Affymetrix 6.0.

As the bench mark, imputation with SNPs available on the 1M array resulted in the highest quality (average $R^2 = 0.91$ in European Americans and average $R^2 = 0.89$ in African Americans). As the number of different arrays increased and the number of genotyped SNPs in the intersecting set decreased, the resulting quality of imputed SNPs also decreased. However, decreases in quality were not as rapid as might be expected. In European Americans (Fig. 3a), imputation quality remained reasonable for input genotyped SNP sets derived from the intersection of up to four Illumina arrays even though only 30 % of the original 1M SNPs were used in the imputation: average $R^2 = 0.79$ across the MAF spectrum and several higher MAF bins having average $R^2 \geq 0.9$. Imputation quality was further reduced with the inclusion of an older Illumina array (HumanHap300-Duo version 2): average $R^2 < 0.9$ for each MAF bin and average $R^2 = 0.71$ across the MAF spectrum. Imputation quality resulting from the intersection of the Illumina 1M and Affymetrix 6.0 arrays was comparable to the imputation quality resulting from the smallest input genotyped SNP set (that is, the largest number of arrays combined among the Illumina arrays), with average $R^2 = 0.73$ across the MAF spectrum. In African Americans (Fig. 3b), poorer imputation quality was observed for all input genotyped SNP sets, as expected, but the relative patterns were similar to those observed in European Americans. The lowest imputation quality in African Americans resulted from the intersection of all five Illumina arrays, with average $R^2 = 0.56$ across the MAF spectrum.

Potential benefits and costs of using public controls

The success in eliminating the bias of imputing SNPs across arrays by using the intersection approach must be balanced with practical considerations of using public controls genotyped on multiple arrays. Two scenarios are most relevant. The first is to consider adding public controls to an existing sample, which increases sample size but necessitates SNP imputation to generate a common set of SNPs for analysis and engenders imputation error that reduces effective sample size. To examine the balance of these two effects on statistical power, we examined a simplified scenario in which a study has 2,000 cases and 2,000 controls genotyped, providing 80 % power to detect an effect size of 1 % variance explained at genome-wide significance ($P \leq 5 \times 10^{-8}$). Figure 4 presents the power estimates by level of imputation accuracy (average R^2) for differing numbers of public controls added to the baseline design. Compared to the baseline model (blue diamond), adding 500 public controls (pink curve) does not improve and may worsen power: showing equivalent power to the baseline model when $R^2 = 0.9$ but steadily declining as imputation accuracy declines. Adding increasing numbers of public controls results in a marginal to substantial improvement in power. For example, adding 2,000 public controls (2,000 cases: 4,000 controls—green line) increases power to between 86 and 93 % when R^2 is between 0.8 and 0.9.

The second scenario to consider for use of public controls is in making decisions about GWAS design: given a budget sufficient to ascertain and genotype 4,000 individuals is the most advantageous power achieved by following this baseline study design (2,000 cases and 2,000 controls) or by reducing the number of study controls ascertained and genotyped, relying on public controls instead? Figure 5 presents power by imputation accuracy for the baseline study design (blue diamond and blue dashed line) and several alternatives. To a much greater extent than adding public controls to an existing study (Fig. 4), redirecting resources to increase cases and relying on public controls appears to substantially increase

NIH-PA Author Manuscript

NIH-PA Author Manuscript

NIH-PA Author Manuscript

power of a study (Fig. 5). Choosing a study design that targets 3,000 cases, 1,000 study controls, and 2,000 public controls increases power to between 85 and 97 % for average R^2 of 0.7–0.9. Pushing this approach further to targeting 4,000 cases and using all public controls makes a more substantial improvement in power [e.g., obtaining 4,000 cases and 8,000 public controls generates greater than 95 % power for average R^2 of 0.5 or greater (purple line)].

Discussion

In this study, we used GWAS data from public repositories to generate common sets of SNPs and to estimate the magnitude of imputation-induced bias among European Americans and African Americans genotyped on different Illumina and Affymetrix arrays. Imputation based on the union of genotyped SNPs available on either the Illumina 1M or 550v3 array showed spurious associations for ~0.2 % of SNPs in both European Americans and African Americans, translating to ~2,000 false positives per one million imputed SNPs. SNPs in low LD regions were more prone to imputation-induced bias, as compared to SNPs in high LD. False positives remained problematic for even very similar arrays (i.e., Illumina 550v1 vs. 550v3), albeit to a lesser extent with 0.07 % of imputed SNPs having spurious association in each ethnic group. False positives were substantial for imputation across array families (Illumina and Affymetrix), amounting to 0.53 and 0.63 % of imputed SNPs (5,000–6,000 false positives per one million imputed SNPs) in European Americans and African Americans, respectively. These results are consistent with Sinnott and Kraft, who estimated an average false positive rate (based on the genome-wide significance threshold of $P < 5 \times 10^{-8}$) of 0.4 % among 2,347,809 imputed SNPs based on a study sample composed of healthy control groups of European descent who were genotyped on Affymetrix 6.0 (subjects arbitrarily designed as cases) or Illumina 550v1 (subjects arbitrarily designated as controls) (Sinnott and Kraft 2012). They observed false positive rates as high as 1.3 % when imputing SNPs genotyped from Illumina but not Affymetrix. Similarly, Uh et al. (2012) reported a genomic control inflation factor well above 1.0 ($\lambda_{gc} = 1.16$), indicative of many false positive associations when imputing across Affymetrix and Illumina arrays. In the current study, there was no evidence of false positive associations among the genotyped SNPs for any pair-wise set of arrays, strongly suggesting that the observed bias among the imputed SNPs is due to the imputation process rather than differences in genotyping quality. Moreover, it is clear from these studies that the degree of bias in a common set of SNPs imputed based on the union of genotyped SNPs from different arrays is too great to permit reliable analyses if left uncorrected.

In both the current study and the Sinnott and Kraft (Sinnott and Kraft 2012) study, SNPs with R^2 >0.3 but exhibiting bias were predominantly SNPs with lower MAF (≤10 %). It is also the case that both studies used HapMap reference panels for imputation. Since 1000 Genomes panels are enriched for lower MAF SNPs, one would expect that imputation based on 1000 Genomes panels would generate greater rates of bias than observed in these studies. Thus, as the field moves forward to take advantage of these more comprehensive reference panels for imputation, correction of this cross array imputation bias will be even more important.

To ameliorate the observed bias, Sinnott and Kraft (2012) tested three methods of correction: (1) use of principal components as covariates in logistic regression analyses; (2) restricting analyses to imputed SNPs with high accuracy, up to $R^2 = 0.99$; and (3) genotyping a subset of controls on the array used for cases to screen out problematic SNPs. Only genotyping a subset of controls provided a level of correction that would avoid a large number of false positive associations (Sinnott and Kraft 2012). However, this correction method is not applicable for use in studies without access to original study DNA or where

NIH-PA Author Manuscript
NIH-PA Author Manuscript
NIH-PA Author Manuscript

budgets would not allow for additional genotyping; both are important limitations when using publicly available genotype data. Uh et al. (2012) proposed a strategy of post-imputation filtering using their R_T^2 statistic ($R_T^2 \geq 0.98$), which was analogous to Sinnott and Kraft's filtering on $R^2 = 0.99$ but for a sibling pair plus control design. Both post-imputation filtering strategies substantially reduced the observed bias in the SNPs meeting their filter requirements, but unacceptable error rates remained (500 false positives for every 1 million imputed SNPs) (Sinnott and Kraft 2012). Moreover, correction strategies based on such highly stringent quality control metrics (i.e., R^2 or R_T^2) require the exclusion of a large number of imputed SNPs from analyses and possibly lead to reduced statistical power and an inability to identify truly associated SNPs, especially in regions with little LD (Uh et al. 2012; Beecham et al. 2010). Applying these stringent exclusions will be particularly problematic for African-derived populations, who have shorter regions of LD across the genome.

In contrast, the strategy proposed in this study, which imputes based on the intersection of SNPs genotyped on all arrays represented in the combined sample, showed no evidence of bias. We are not aware of any other method that eliminates the imputation-induced bias without some study samples being simultaneously genotyped on all the arrays being used for imputation. Estimating from more information is generally expected to provide better statistical estimates than estimating from less. For this reason, imputation using the union of SNPs available across the genotyping arrays in the studies to be combined could be expected to produce the best imputation results. However, it is known that differing haplotype information quality generates differences in imputation accuracy (Almeida et al. 2011). Extending this observation to different arrays across which there are differing amounts of genetic information (i.e., numbers of SNPs) or differing types of genetic information (i.e., differing SNP selection strategies used for Illumina and Affymetrix), one would expect differing imputation accuracy results from the different arrays. Combining imputation across arrays with differing inputs seems likely to generate systematically differential imputation error among individuals contributing to the composite data set and thus the observed biases, including greater bias among SNPs with lower MAF, for which genetic information on which to base prediction of imputed SNPs is less.

The limiting factor of our intersection-of-SNPs strategy for imputing to a common set of SNPs from different arrays is the degree of overlap in genotyped SNPs present across the arrays to be combined. Using the R^2 statistic as a measure of imputation quality, this study demonstrated decreasing imputation quality as the number of overlapping genotyped SNPs decreased. However, this effect did not appear to be as dramatic as might be expected. For example, the overlap between the Illumina 1M and 550v3 arrays on chr.22 was ~7,900 SNPs out of the ~14,000 SNPs on the 1M array (56 % of the original number of SNPs), but the reduction in average imputation quality without any filtering was modest ($R^2 = 0.91$ vs. 0.88 in European American and $R^2 = 0.89$ vs. 0.83 in African American). The intersection strategy remained viable even when including several arrays; imputation based on ~4,000 overlapping genotyped SNPs across the Illumina 1M, Omni1-Quad, 660W, and 550v3 (~30 % of the ~14,000 on the 1M array for chr.22) showed an average $R^2 = 0.79$ across the MAF spectrum for European Americans and $R^2 = 0.68$ for African Americans. Because of the differing SNP selection strategies, the overlap between the Illumina 1M and Affymetrix 6.0 was low (~3,200 SNPs), resulting in somewhat poorer imputation quality: $R^2 = 0.73$ in European Americans and $R^2 = 0.61$ in African Americans. A related consideration may be the number and character of SNPs being imputed. In this study's examples, the number of genotyped SNPs changes as one adds arrays to the intersection, but the number of imputed SNPs remains the same. In parallel, it may be that keeping the number of genotyped SNPs the same but increasing the number of imputed SNPs will reduce imputation accuracy as the genotyped SNPs will likely have weaker correlations with the larger set of imputed SNPs

NIH-PA Author Manuscript
NIH-PA Author Manuscript
NIH-PA Author Manuscript

and their characteristics change. We have observed this in another study testing differences in imputation performance among African Americans by reference panels and imputation software (Hancock et al. 2012). In that study, the same genotyped SNPs were used for imputation with both HapMap and 1000G reference panels, but the average R^2 was reduced somewhat for 1000G due to the greater prevalence of low MAF SNPs in the 1000G panels. The counter point is that the coverage for 1000G was much better. Thus, investigators must balance imputation accuracy and coverage in choosing which approach to take.

Filtering SNPs based on imputation quality metrics (e.g., $R^2 < 0.3$) prior to GWAS analysis is not recommended because of the potential to miss true associations (Beecham et al. 2010). Instead, substantiating imputed SNP associations requires quality assessment and replication testing in independent studies, both of which will be even more important for data imputed across arrays.

As a precursor to the aforementioned imputation-based strategies, Mukherjee et al. (2011) investigated combining control samples for only those SNPs genotyped on all arrays to be combined. They showed that combining publically available data sets based on only the SNPs shared across arrays is a non-biased technique that can substantially improve statistical power as the control:case ratio increases as long as ancestry stratification and MAF variation across control data sets are properly accounted for (Mukherjee et al. 2011). However, this approach substantially limits coverage of the genome which is likely to reduce statistical power through limited opportunities to test variants at or associated with causal loci (Spencer et al.2009).

The success in eliminating the bias of imputing SNPs across arrays by using the intersection approach brought to the fore practical considerations of when use of public controls genotyped on multiple arrays is worthwhile. Thus, in a final set of analyses, we examined the effects of increasing sample size and reducing imputation accuracy on statistical power when using public controls under two simplified scenarios. First, adding public controls to an existing genotyped sample of cases and controls meaningfully increased power with the addition of as little as one-third of the original control sample if imputation accuracy remained moderate to good (average $R^2 \geq 0.7$). Second, designing a GWAS to rely on public controls to supplement or replace study controls showed even more marked increases in statistical power by focusing fixed resources on increasing the number of cases genotyped as well as boosting the size of the control group.

These power scenarios suggest there are many cases in which using public controls in GWAS would substantially improve power and justify the additional effort to obtain and use public controls. However, they do not take into account additional issues with use of public controls including differences in phenotype measurement, as well as potential systematic genetic and environmental differences between the public controls and study participants. Use of public controls requires either phenotype harmonization across contributing samples or, alternatively, use of population controls where the phenotype is not measured but is rare enough in the population that misclassification of true cases as controls is unlikely. Similarly, careful attention to population stratification across contributing public control datasets or between case and control datasets is also necessary to ensure that systematic biases are not introduced into the analyses of the combined datasets. For example, ascertaining African American study participants from one part of the United States and obtaining African American public controls ascertained from another location could introduce systematic differences and spurious findings due to differing types/levels of admixture or differences in environmental risks. Thus, the potential benefit of using public controls in terms of improving statistical power due to increased sample size must be weighed in each particular circumstance against the increased complexity of analyses and

NIH-PA Author Manuscript

NIH-PA Author Manuscript

NIH-PA Author Manuscript

the potential loss of power due to poor imputation or other systematic problems arising from differently recruited cases and controls.

Imputation of untyped SNPs has become an important tool for discovery of new genotype-phenotype associations, generally improving density of coverage and statistical power (Spencer et al. 2009). Extending SNP imputation tools to the context of generating a common set of SNPs for analysis of samples genotyped on different arrays has proved challenging, with substantial biases observed here and in prior studies (Sinnott and Kraft 2012; Uh et al. 2012). However, the promise of accurately conducting this type of imputation is to substantially extend the benefit of publicly sharing GWAS data through repositories like dbGaP. Combining the original phenotypic and genotypic data from several studies into a single population control group and pairing these combined data with cases of the phenotype of interest allow for powerful opportunities to identify new genetic associations. A composite set of public controls can also be used to augment study controls to increase sample size and boost statistical power (Ho and Lange 2010). These study designs extend the scientific and societal benefits from the financial and time investments made by the original studies' funding agencies and investigators, providing a cost-effective strategy to obtain the large number of control subjects needed for GWAS analyses. This strategy may be particularly beneficial for ancestry groups with few available samples (e.g., African Americans) (Hartz et al. 2011). Thus, continued examination and development of methods to produce valid SNP imputation across historic and new genotyping arrays is well worth the investment. Use of the intersecting SNP strategy described in this study appears to be a cost-effective and valid approach to cross array imputation, avoiding previously observed biases and generating reasonable imputation quality across arrays with 30 % or more overlap in genotyped SNPs.

Supplementary Material

Refer to Web version on PubMed Central for supplementary material.

Acknowledgments

This work was supported by National Institute of Drug Abuse grant nos. R33DA027486 and R01DA026141 (E.O. Johnson PI), as well as R01DA025888 (L.J. Bierut & E.O. Johnson Co-PIs). Funding support for SAGE was provided through the NIH Genes, Environment and Health Initiative [GEI] (U01 HG004422). SAGE is one of the GWAS funded as part of the Gene Environment Association Studies (GENEVA) under GEI. Assistance with phenotype harmonization and genotype cleaning, as well as with general study coordination, was provided by the GENEVA Coordinating Center (U01 HG004446). Assistance with data cleaning was provided by the National Center for Biotechnology Information. Support for collection of datasets and samples was provided by the Collaborative Study on the Genetics of Alcoholism (COGA; U10 AA008401), the Collaborative Genetic Study of Nicotine Dependence (COGEND; P01 CA089392), and the Family Study of Cocaine Dependence (FSCD; R01 DA013423). Funding support for genotyping, which was performed at the Johns Hopkins University Center for Inherited Disease Research, was provided by the NIH GEI (U01HG004438), the National Institute on Alcohol Abuse and Alcoholism, the National Institute on Drug Abuse, and the NIH contract "High throughput genotyping for studying the genetic contributions to human disease" (HHSN268200782096C). The SAGE dataset used for the analyses described in this manuscript was obtained from dbGaP at http://www.ncbi.nlm.nih.gov/projects/gap/, through accession number phs000092.v1.p1. The CGEMS (http://cgems.cancer.gov/) PanScan study was derived from 12 cohorts, as outlined by Amundadottir et al. (2009). The PanScan dataset used for the analyses described in this manuscript was obtained from dbGaP through accession number phs000206.v3.p2. The CGEMS breast cancer GWAS was derived from the Nurses' Health Study, which was supported by NIH grants CA65725, CA87969, CA49449, CA67262, CA50385, and 5UO1 CA098233. The CGEMS dataset used for the analyses described in this manuscript was obtained from dbGaP through accession number phs000147.v1.p1. Funding support for the GWAS of Schizophrenia was provided by the National Institute of Mental Health (R01 MH67257, R01 MH59588, R01 MH59571, R01 MH59565, R01 MH59587, R01 MH60870, R01 MH59566, R01 MH59586, R01 MH61675, R01 MH60879, R01 MH81800, U01 MH46276, U01 MH46289 U01 MH46318, U01 MH79469, and U01 MH79470), and the genotyping of samples was provided through GAIN. The datasets used for the analyses described in this manuscript were obtained from the dbGaP through accession number phs000021.v3.p2. Samples and associated phenotype data for the GWAS of Schizophrenia were provided by the Molecular Genetics of Schizophrenia

Collaboration (PI: Pablo V. Gejman, Evanston Northwestern Healthcare (ENH) and Northwestern University, Evanston, IL, USA).

References

Almeida MA, Oliveira PS, Pereira TV, Krieger JE, Pereira AC. An empirical evaluation of imputation accuracy for association statistics reveals increased type-I error rates in genome-wide associations. BMC Genet. 2011; 12:10. doi: 10.1186/1471-2156-12-10. [PubMed: 21251252]

Altshuler DM, Gibbs RA, Peltonen L, Dermitzakis E, Schaffner SF, Yu F, Bonnen PE, de Bakker PI, Deloukas P, Gabriel SB, Gwilliam R, Hunt S, Inouye M, Jia X, Palotie A, Parkin M, Whittaker P, Chang K, Hawes A, Lewis LR, Ren Y, Wheeler D, Muzny DM, Barnes C, Darvishi K, Hurles M, Korn JM, Kristiansson K, Lee C, McCarrol SA, Nemesh J, Keinan A, Montgomery SB, Pollack S, Price AL, Soranzo N, Gonzaga-Jauregui C, Anttila V, Brodeur W, Daly MJ, Leslie S, McVean G, Moutsianas L, Nguyen H, Zhang Q, Ghori MJ, McGinnis R, McLaren W, Takeuchi F, Grossman SR, Shlyakhter I, Hostetter EB, Sabeti PC, Adebamowo CA, Foster MW, Gordon DR, Licinio J, Manca MC, Marshall PA, Matsuda I, Ngare D, Wang VO, Reddy D, Rotimi CN, Royal CD, Sharp RR, Zeng C, Brooks LD, McEwen JE. Integrating common and rare genetic variation in diverse human populations. Nature. 2010; 467(7311):52–58. doi: 10.1038/nature09298. [PubMed: 20811451]

Amundadottir L, Kraft P, Stolzenberg-Solomon RZ, Fuchs CS, Petersen GM, Arslan AA, Bueno-de-Mesquita HB, Gross M, Helzlsouer K, Jacobs EJ, LaCroix A, Zheng W, Albanes D, Bamlet W, Berg CD, Berrino F, Bingham S, Buring JE, Bracci PM, Canzian F, Clavel-Chapelon F, Clipp S, Cotterchio M, de Andrade M, Duell EJ, Fox JW Jr, Gallinger S, Gaziano JM, Giovannucci EL, Goggins M, Gonzalez CA, Hallmans G, Hankinson SE, Hassan M, Holly EA, Hunter DJ, Hutchinson A, Jackson R, Jacobs KB, Jenab M, Kaaks R, Klein AP, Kooperberg C, Kurtz RC, Li D, Lynch SM, Mandelson M, McWilliams RR, Mendelsohn JB, Michaud DS, Olson SH, Overvad K, Patel AV, Peeters PH, Rajkovic A, Riboli E, Risch HA, Shu XO, Thomas G, Tobias GS, Trichopoulos D, Van Den Eeden SK, Virtamo J, Wactawski-Wende J, Wolpin BM, Yu H, Yu K, Zeleniuch-Jacquotte A, Chanock SJ, Hartge P, Hoover RN. Genome-wide association study identifies variants in the ABO locus associated with susceptibility to pancreatic cancer. Nat Genet. 2009; 41(9):986–990. doi: 10.1038/ng.429. [PubMed: 19648918]

Beecham GW, Martin ER, Gilbert JR, Haines JL, Pericak-Vance MA. APOE is not associated with Alzheimer disease: a cautionary tale of genotype imputation. Ann Hum Genet. 2010; 74(3):189–194. doi: 10.1111/j.1469-1809.2010.00573.x. [PubMed: 20529013]

Bierut LJ, Agrawal A, Bucholz KK, Doheny KF, Laurie C, Pugh E, Fisher S, Fox L, Howells W, Bertelsen S, Hinrichs AL, Almasy L, Breslau N, Culverhouse RC, Dick DM, Edenberg HJ, Foroud T, Grucza RA, Hatsukami D, Hesselbrock V, Johnson EO, Kramer J, Krueger RF, Kuperman S, Lynskey M, Mann K, Neuman RJ, Nothen MM, Nurnberger JI Jr, Porjesz B, Ridinger M, Saccone NL, Saccone SF, Schuckit MA, Tischfield JA, Wang JC, Rietschel M, Goate AM, Rice JP. A genome-wide association study of alcohol dependence. Proc Natl Acad Sci USA. 2010; 107(11):5082–5087. doi: 10.1073/pnas.0911109107. [PubMed: 20202923]

Durbin RM, Abecasis GR, Altshuler DL, Auton A, Brooks LD, Gibbs RA, Hurles ME, McVean GA. A map of human genome variation from population-scale sequencing. Nature. 2010; 467(7319):1061–1073. doi: 10.1038/nature09534. [PubMed: 20981092]

Fellay J, Shianna KV, Ge D, Colombo S, Ledergerber B, Weale M, Zhang K, Gumbs C, Castagna A, Cossarizza A, Cozzi-Lepri A, De Luca A, Easterbrook P, Francioli P, Mallal S, Martinez-Picado J, Miro JM, Obel N, Smith JP, Wyniger J, Descombes P, Antonarakis SE, Letvin NL, McMichael AJ, Haynes BF, Telenti A, Goldstein DB. A whole-genome association study of major determinants for host control of HIV-1. Science. 2007; 317(5840):944–947. doi: 10.1126/science.1143767. [PubMed: 17641165]

Hancock DB, Levy JL, Gaddis NC, Bierut LJ, Saccone NL, Page GP, Johnson EO. Assessment of genotype imputation performance using 1000 Genomes in African American studies. PLoS ONE. 2012; 7(11):e50610. doi: 10.1371/journal.pone.0050610. [PubMed: 23226329]

Hartz SM, Johnson EO, Saccone NL, Hatsukami D, Breslau N, Bierut LJ. Inclusion of African Americans in genetic studies: what is the barrier? Am J Epidemiol. 2011; 174(3):336–344. doi: 10.1093/aje/kwr084. [PubMed: 21633120]

NIH-PA Author Manuscript NIH-PA Author Manuscript NIH-PA Author Manuscript

NIH-PA Author Manuscript NIH-PA Author Manuscript NIH-PA Author Manuscript

Ho LA, Lange EM. Using public control genotype data to increase power and decrease cost of case-control genetic association studies. Hum Genet. 2010; 128(6):597–608. doi: 10.1007/s00439-010-0880-x. [PubMed: 20821337]

Howie B, Marchini J, Stephens M. Genotype imputation with thousands of genomes. G3 (Bethesda). 2011; 1(6):457–470. doi: 10.1534/g3.111.001198. [PubMed: 22384356]

Hunter DJ, Kraft P, Jacobs KB, Cox DG, Yeager M, Hankinson SE, Wacholder S, Wang Z, Welch R, Hutchinson A, Wang J, Yu K, Chatterjee N, Orr N, Willett WC, Colditz GA, Ziegler RG, Berg CD, Buys SS, McCarty CA, Feigelson HS, Calle EE, Thun MJ, Hayes RB, Tucker M, Gerhard DS, Fraumeni JF Jr, Hoover RN, Thomas G, Chanock SJ. A genome-wide association study identifies alleles in FGFR2 associated with risk of sporadic postmenopausal breast cancer. Nat Genet. 2007; 39(7):870–874. doi: 10.1038/ng2075. [PubMed: 17529973]

Li Y, Willer CJ, Ding J, Scheet P, Abecasis GR. MaCH: using sequence and genotype data to estimate haplotypes and unobserved genotypes. Genet Epidemiol. 2010; 34(8):816–834. doi: 10.1002/gepi.20533. [PubMed: 21058334]

Manichaikul A, Mychaleckyj JC, Rich SS, Daly K, Sale M, Chen WM. Robust relationship inference in genome-wide association studies. Bioinformatics. 2010; 26(22):2867–2873. doi: 10.1093/bioinformatics/btq559. [PubMed: 20926424]

Manolio TA, Rodriguez LL, Brooks L, Abecasis G, Ballinger D, Daly M, Donnelly P, Faraone SV, Frazer K, Gabriel S, Gejman P, Guttmacher A, Harris EL, Insel T, Kelsoe JR, Lander E, McCowin N, Mailman MD, Nabel E, Ostell J, Pugh E, Sherry S, Sullivan PF, Thompson JF, Warram J, Wholley D, Milos PM, Collins FS. New models of collaboration in genome-wide association studies: the Genetic Association Information Network. Nat Genet. 2007; 39(9):1045–1051. doi: 10.1038/ng2127. [PubMed: 17728769]

Marchini J, Howie B. Genotype imputation for genome-wide association studies. Nat Rev Genet. 2010; 11(7):499–511. doi: 10.1038/nrg2796. [PubMed: 20517342]

Mukherjee S, Simon J, Bayuga S, Ludwig E, Yoo S, Orlow I, Viale A, Offit K, Kurtz RC, Olson SH, Klein RJ. Including additional controls from public databases improves the power of a genome-wide association study. Hum Hered. 2011; 72(1):21–34. doi: 10.1159/000330149. [PubMed: 21849791]

Pasaniuc B, Rohland N, et al. Extremely low-coverage sequencing and imputation increases power for genome-wide association studies. Nat Genet. 2012; 44(6):631–635. [PubMed: 22610117]

Price AL, Patterson NJ, Plenge RM, Weinblatt ME, Shadick NA, Reich D. Principal components analysis corrects for stratification in genome-wide association studies. Nat Genet. 2006; 38(8): 904–909. doi: 10.1038/ng1847. [PubMed: 16862161]

Pritchard JK, Stephens M, Donnelly P. Inference of population structure using multilocus genotype data. Genetics. 2000; 155(2):945–959. [PubMed: 10835412]

Pritchard JK, Przeworski M. Linkage disequilibrium in humans: models and data. Am J Hum Genet. 2001; 69(1):1–14. [PubMed: 11410837]

Purcell S, Neale B, Todd-Brown K, Thomas L, Ferreira MA, Bender D, Maller J, Sklar P, de Bakker PI, Daly MJ, Sham PC. PLINK: a tool set for whole-genome association and population-based linkage analyses. Am J Hum Genet. 2007; 81(3):559–575. doi: 10.1086/519795. [PubMed: 17701901]

Shriner D, Adeyemo A, Chen G, Rotimi CN. Practical considerations for imputation of untyped markers in admixed populations. Genet Epidemiol. 2010; 34(3):258–265. doi: 10.1002/gepi.20457. [PubMed: 19918757]

Sinnott JA, Kraft P. Artifact due to differential error when cases and controls are imputed from different platforms. Hum Genet. 2012; 131(1):111–119. doi: 10.1007/s00439-011-1054-1. [PubMed: 21735171]

Southam L, Panoutsopoulou K, Rayner NW, Chapman K, Durrant C, Ferreira T, Arden N, Carr A, Deloukas P, Doherty M, Loughlin J, McCaskie A, Ollier WE, Ralston S, Spector TD, Valdes AM, Wallis GA, Wilkinson JM, Marchini J, Zeggini E. The effect of genome-wide association scan quality control on imputation outcome for common variants. Eur J Hum Genet. 2011; 19(5):610–614. doi: 10.1038/ejhg.2010.242. [PubMed: 21267008]

NIH-PA Author Manuscript
NIH-PA Author Manuscript
NIH-PA Author Manuscript

Spencer CC, Su Z, Donnelly P, Marchini J. Designing genome-wide association studies: sample size, power, imputation, and the choice of genotyping chip. PLoS Genet. 2009; 5(5):e1000477. doi: 10.1371/journal.pgen.1000477. [PubMed: 19492015]

Tiwari HK, Birkner T, et al. Accurate and flexible power calculations on the spot: applications to genomic research. Stat Interface. 2011; 4(3):353–358. [PubMed: 22022634]

Uh HW, Deelen J, Beekman M, Helmer Q, Rivadeneira F, Hottenga JJ, Boomsma DI, Hofman A, Uitterlinden AG, Slagboom PE, Bohringer S, Houwing-Duistermaat JJ. How to deal with the early GWAS data when imputing and combining different arrays is necessary. Eur J Hum Genet. 2012; 20(5):572–576. doi: 10.1038/ejhg.2011.231. [PubMed: 22189269]

Zheng J, Li Y, et al. A comparison of approaches to account for uncertainty in analysis of imputed genotypes. Genet Epidemiol. 2011; 35(2):102–110. [PubMed: 21254217]

Zhuang JJ, Zondervan K, Nyberg F, Harbron C, Jawaid A, Cardon LR, Barratt BJ, Morris AP. Optimizing the power of genome-wide association studies by using publicly available reference samples to expand the control group. Genet Epidemiol. 2010; 34(4):319–326. [PubMed: 20088020]

NIH-PA Author Manuscript
NIH-PA Author Manuscript
NIH-PA Author Manuscript

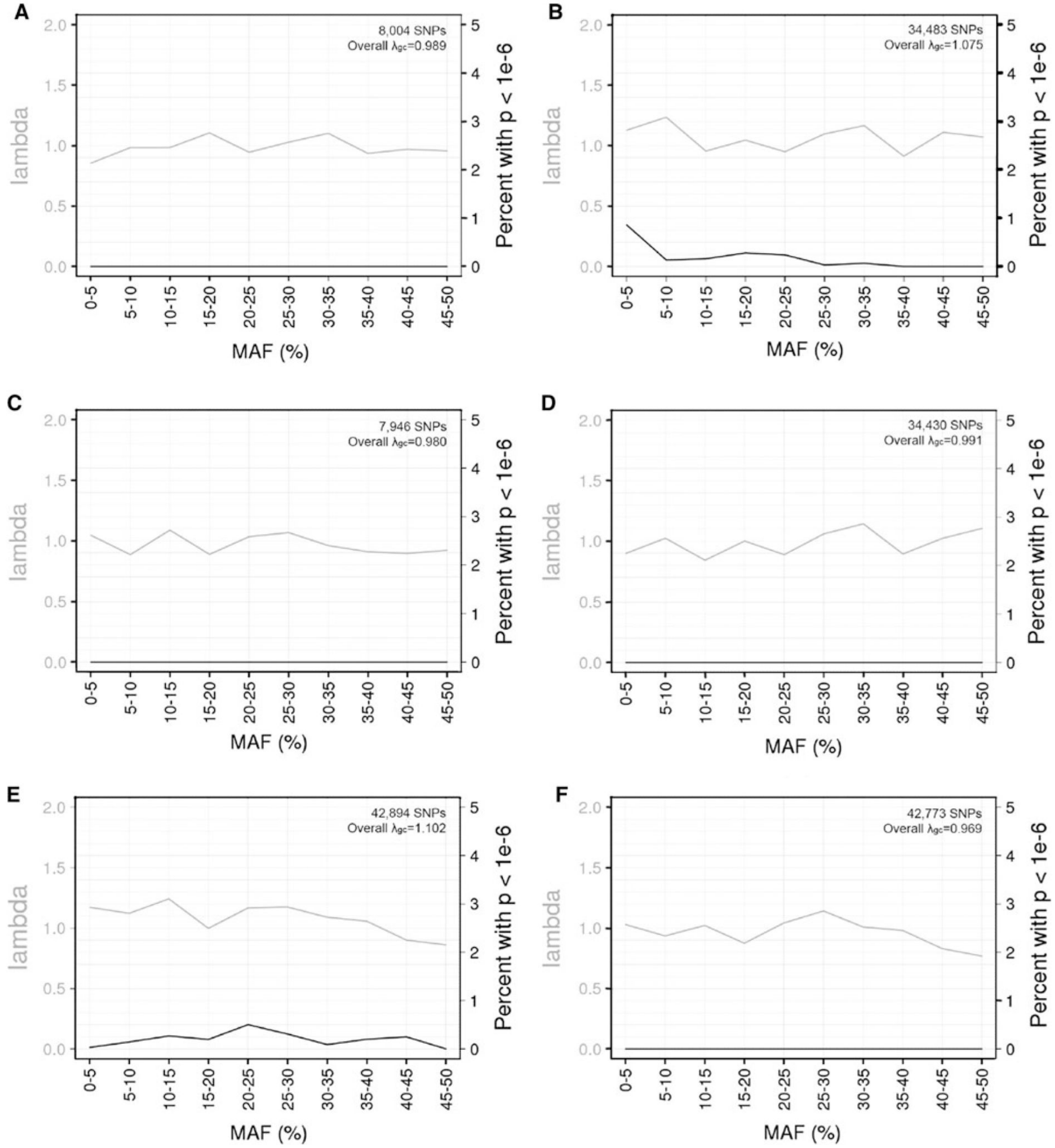

Fig. 1.

Genomic inflation factors (*grey lines*) (λ_{gc}) and percentages of SNPs having spurious association (*black lines*) ($P < 1 \times 10^{-6}$), by minor allele frequency (MAF), when combining studies genotyped on different Illumina BeadChip arrays (Human1M or HumanHap550 version 3). **a–c** European American subjects from SAGE were compared to PanScan subjects, and **d-f** African American subjects from SAGE were compared to iControl subjects. Three different SNP sets were assessed: **a, d** genotyped SNPs available on both arrays; **b, e** imputed SNPs based on the union of genotyped SNPs available on either array;

and **c, f** imputed SNPs based on the intersection of genotyped SNPs available on both arrays. The number of SNPs with MAF >1 % and the overall λ_{gc} are shown in each plot

NIH-PA Author Manuscript
NIH-PA Author Manuscript
NIH-PA Author Manuscript

NIH-PA Author Manuscript

NIH-PA Author Manuscript

NIH-PA Author Manuscript

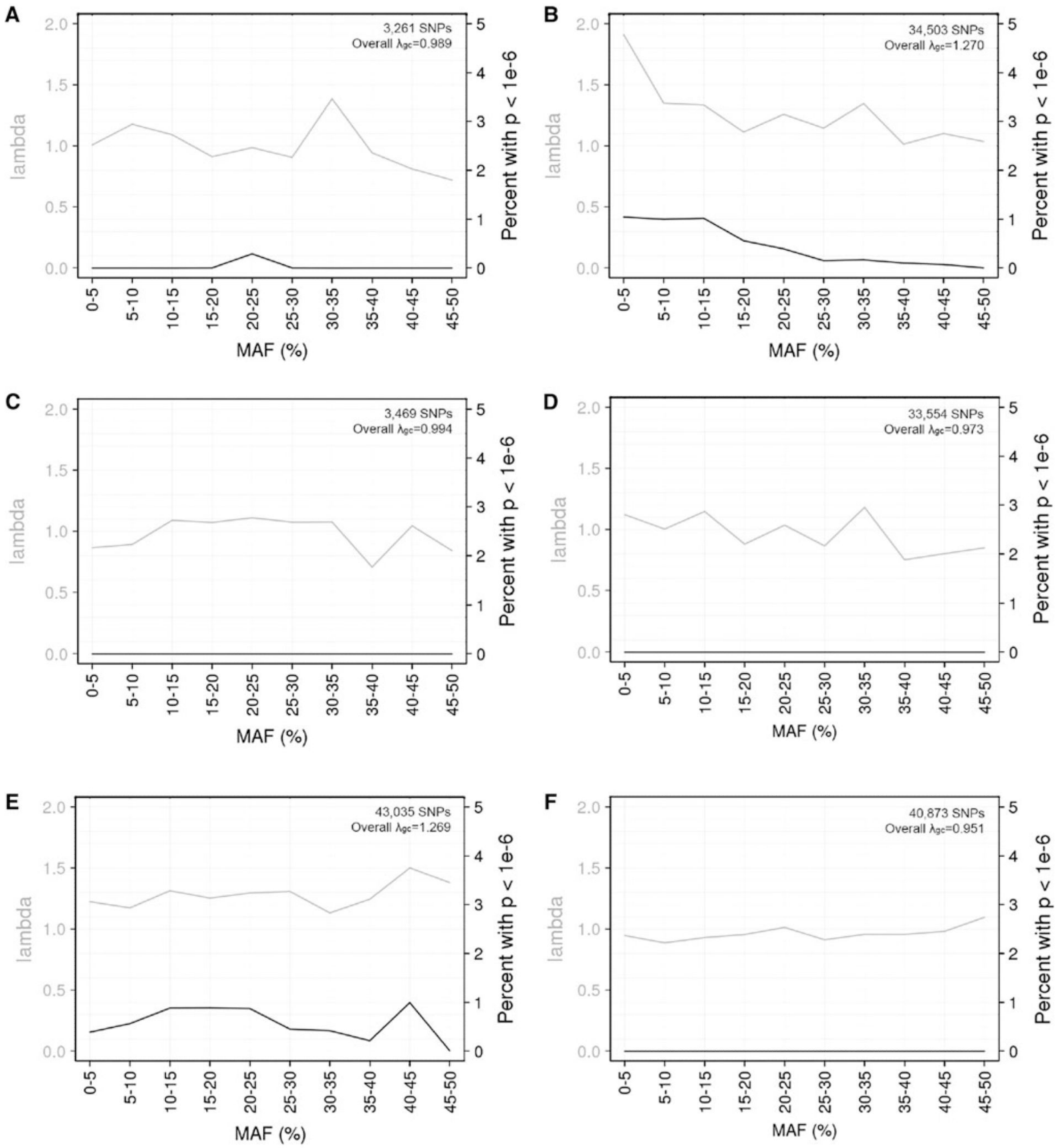

Fig. 2.

Genomic inflation factors (*grey lines*) (λ_{gc}) and percentages of SNPs having spurious association (*black lines*) ($P < 1 \times 10^{-6}$), by minor allele frequency (MAF), when combining studies genotyped on either the Illumina Human1M or Affymetrix 6.0 array. **a–c** European American and **d–f** African American subjects from SAGE (genotyped on Illumina 1M) were compared to subjects from the GAIN GWAS of Schizophrenia (genotyped on Affymetrix 6.0). Three different SNP sets were assessed: **a, d** genotyped SNPs available on both arrays; **b, e** imputed SNPs based on the union of genotyped SNPs available on either array; and **c, f**

NIH-PA Author Manuscript

NIH-PA Author Manuscript

NIH-PA Author Manuscript

imputed SNPs based on the intersection of genotyped SNPs available on both arrays. The number of SNPs with MAF >1 % and the overall λ_{gc} are shown in each plot

NIH-PA Author Manuscript
NIH-PA Author Manuscript
NIH-PA Author Manuscript

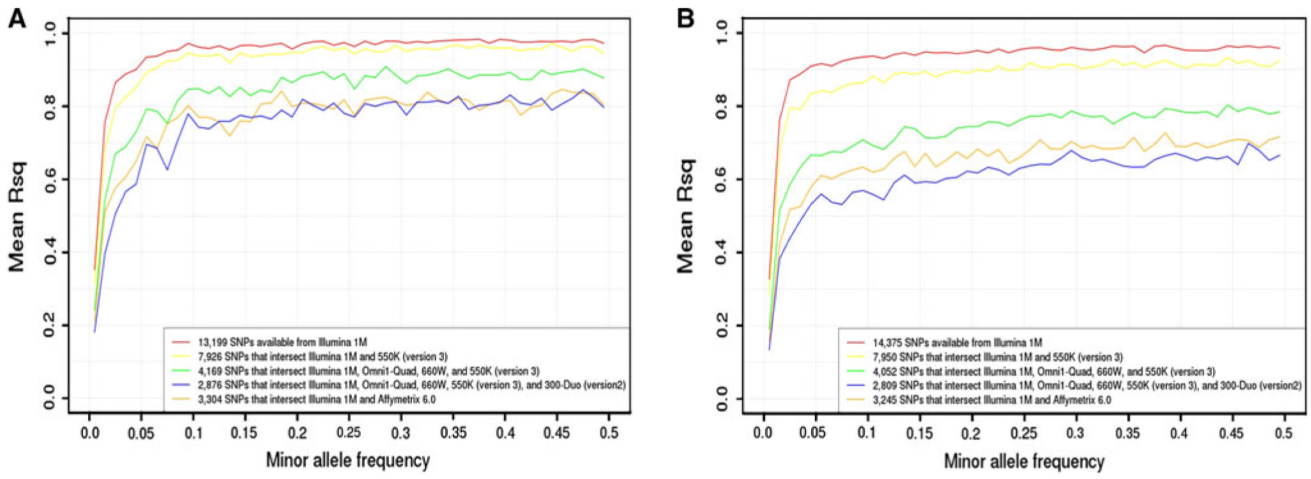

Fig. 3.

Average R^2 values in SAGE control subjects (genotyped on Illumina's Human1M) to indicate overall quality across all imputed SNPs, when imputation was based on all genotyped SNPs or the intersection of genotyped SNPs with Affymetrix 6.0 or varying Illumina arrays (Human1M, HumanOmni1-Quad, Human660W, HumanHap550 version 1, and HumanHap300-Duo version 2 BeadChip). Results are shown across minor allele frequency (MAF) intervals of 1 % for all imputed SNPs with MAF >1 % on chromosome 22: **a** ~34,000 SNPs in European Americans and **b** ~43,000 SNPs in African Americans

NIH-PA Author Manuscript
NIH-PA Author Manuscript
NIH-PA Author Manuscript

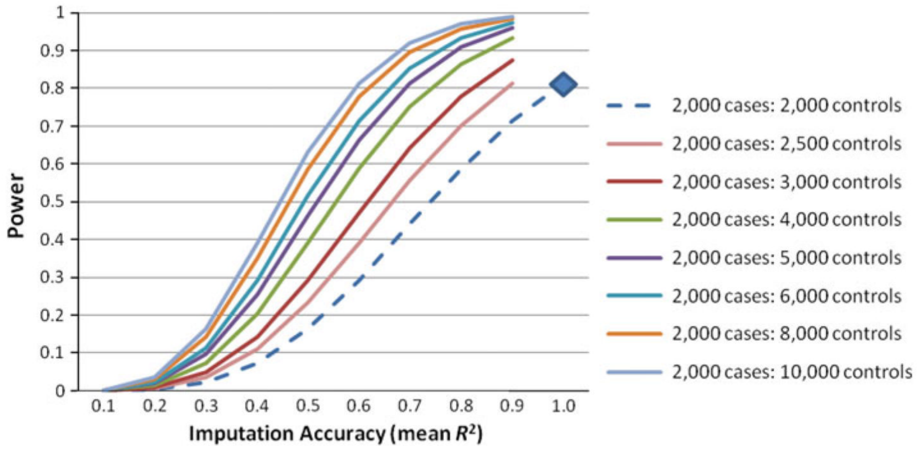

Fig. 4.

Expected statistical power by level of imputation accuracy (average R^2) for differing numbers of public controls added to the baseline design of 2,000 cases and 2,000 controls (*blue diamond* and *blue dashed line*). Power was estimated for detection of a SNP effect size of 1 % explained variance in the phenotype. The baseline model provided 81 % power to detect this effect size at a genome-wide significance of $P = 5 \times 10^{-8}$

NIH-PA Author Manuscript
NIH-PA Author Manuscript
NIH-PA Author Manuscript

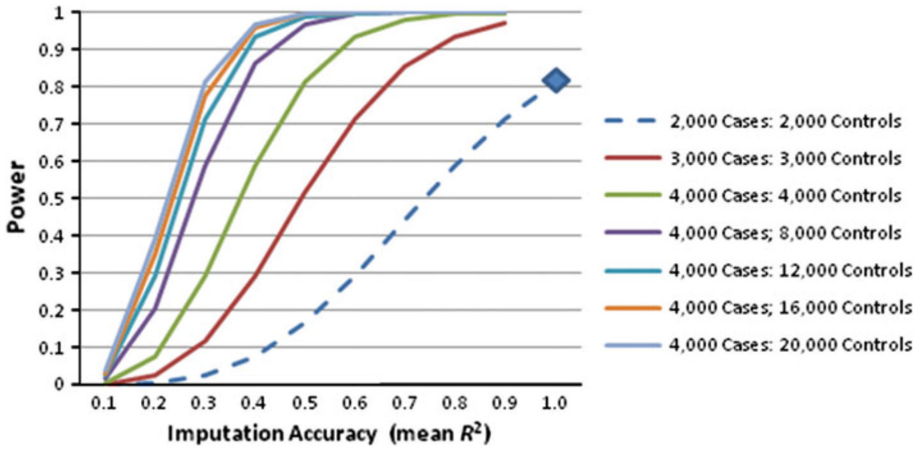

Fig. 5.

Expected statistical power by imputation accuracy (average R^2) for the baseline study design (2,000 cases and 2,000 controls: *blue diamond* and *blue dashed line*) and several alternatives focusing study recruitment and genotyping on increasing numbers of cases and relying on public controls under the constraint of maximal recruitment and genotyping of 4,000 individuals. The baseline model provided 81 % power to detect this effect size at a genome-wide significance of $P = 5 \times 10^{-8}$

NIH-PA Author Manuscript NIH-PA Author Manuscript NIH-PA Author Manuscript

Table 1

Genotyped study subjects and SNPs used for imputation

Ancestry group	Data source[a] (originating study, if applicable)	Illumina genotyping array	No. of genotyped subjects[b]		No. of genotyped SNPs on chromosome 22	
			Before QC	After QC	Before QC	After QC
European American	dbGaP (SAGE, Bierut et al. 2010)	Human1M	1,397	1,360	16,047	13,199
	dbGap (PanScan, Amundadottir et al. 2009)	HumanHap550 (version 3)	1,897	1,783	8,462	8,042
	dbGaP (CGEMS breast cancer GWAS, Hunter et al. 2007)	HumanHap550 (version 1)	1,142	1,131	8,229	7,916
	dbGaP (GAIN GWAS of Schizophrenia, Manolio et al. 2007)	Affymetrix 6.0	1,378	1,164	9,347	9,302
African American	dbGaP (SAGE, Bierut et al. 2010)	Human1M	504	431	16,047	14,375
	iControl	HumanHap550 (version 3)	830	595	8,462	8,101
	iControl	HumanHap550 (version 1)	1,331	1,046	8,205	7,920
	dbGaP (GAIN GWAS of Schizophrenia, Manolio et al. 2007)	Affymetrix 6.0	949	693	10,752	10,681

CGEMS cancer genetic markers of susceptibility, *dbGaP* database of genotypes and phenotypes, *GAIN* genetic association information network, *GWAS* genome-wide association study, *PanScan* pancreatic cancer cohort consortium, *QC* quality control, *SAGE* study of addiction: genetics and environment, *SNP* single nucleotide polymorphism

[a]Data from the dbGaP studies were downloaded between June 1, 2011 and June 20, 2011. Data from the iControl database were downloaded on January 19, 2011

[b]Subjects classified as cases in the original dbGaP studies were excluded prior to quality controls to avoid the potential for identifying true genetic differences between disease + cases and disease − controls. This exclusion was not applicable for the iControl subjects
