# Supplementary material for: Identification of common predisposing loci to hematopoietic cancers in four dog breeds
Source: PLoS Genet. 2021 Apr 1;17(4):e1009395. doi: 10.1371/journal.pgen.1009395 (PMC8016107; doi:10.1371/journal.pgen.1009395)
Supplement: Attachment — Submitted filename: reponse-point-by-point _final.docx [file pgen.1009395.s015.docx]

Dear Editor and reviewers

First of all, we thank you for your time to review our work. We appreciate the points raised by the reviewers and they clearly allowed us to improve the quality and presentation of the manuscript.

Based on these comments, we have conducted a more stringent analysis by re-analyzing all GWAS data. To answer to reviewer 3 (minor comment 7), more stringent criteria were used to requalify control dogs; explaining the changes of number of dogs in the article. In addition, we have included, in this new version, a validation analysis of the best SNVs using independent Bernese MountainDog cohorts (1^st^ cohort of 186 cases and 176 controls; 2^nd^ cohort of 317 unaffected dogs with an age <10 years).

Finally, we have re-written the manuscript in order to increase readability and used the English” Taylor & Francis Editing” language editing service. All consequent changes are highlighted in yellow in the main text.

We have also addressed all the specific issues raised by the three reviewers and have made a point by point response (in blue) as follows.

We thank you again for your thoughtful suggestions, indeed the manuscript has strongly benefited from these insightful remarks.

**Reviewer #1:**

Major comments.

«  I confess to having a lot of difficulty in following the author’s presentation. After struggling I ended up questioning the logical order the authors use to tell their story. The progression of 7 GWA with adding breeds and cancer types using just the 173K SNP data was really a load to try to understand and the presentation in general did not help. Having to look for Tables 1 – 3, while the Figures were inserted in the text did not help. 

I suggest that the authors consider starting off right at the clearest analysis and the ones with the most strength; i.e., the large population sizes and imputed data from several lo density arrays on HS alone (GWA 8) that leads to Figure 6. And follow it with the HS GWA that adds in FCR and Rottweilers (GWA 9 and 10). With that strongest and clearest result in hand it seems that some of the figures and analyses with lower density SNPs leading up to it may not be necessary. And then go on to the stories of combining the HS with MCT and Lymphoma. »

We agree that the presentation of the 7 GWAS combing several breeds and several cancers can be difficult to follow. Thus we fully understand the reviewer suggestion to focus on the GWAS with most strength, largest population sizes and imputed data, will certainly clarify the story and we took it into account. However, the GWAS combining HS with MCT and Lymphoma also allowed us to demonstrate that loci linked to cancer in dogs could be shared by several breeds with different cancer types. Moreover, since we do not have Golden retrievers genotyped on high density arrays, we are not able to impute the publicly available genotypes of MCT and Lymphoma on the larger density array. For these reasons, we decided to present the GWAS in the following order :

1- the Histiocytic sarcoma GWAS on Bernese mountain dog using the 170K
2- the combination of HS with Mast cell tumor and lymphoma

These first two paragraphs thus presenting that loci can be shared by several breeds with different cancers.

3- large population sizes and the imputed data from several low density arrays on HS alone (ex GWAS 8) that leads to ex Figure 6.
4- HS GWAS on BMD with the addition of FCR and Rottweilers (ex GWAS 9 and 10)

Moreover we introduced and clarified, in the first paragraph of the results, the numbers of dogs (cases and controls), the breed and the cancer used in each analysis (Cf Table 1).

The pedigree of Figure 2 showing co-segregation of cancers may not tell the whole story. Conclusions on a single pedigree can be influenced by ascertainment bias. And, how many of the dogs have more than one form of cancer? Can the numbers of dogs with each or both conditions across the entire population be provided instead? Anyway I suggest moving Figure 2 to Supplemental.

As suggested, we moved Figure 2 to supplementary data. We do not have the numbers of dogs with each or both conditions across the entire population. Some dogs are affected by mast cell tumor and HS or lymphoma. In the 2005 BMDCA Survey ^1^, it was reported that a majority (~66.5%) of BMDs succumb of cancer and in this survey, the most frequent reported cancer was HS, then mast cell tumors (4%) and then lymphomas. We added in the text the following sentence «

Lanes 138-141: “This hypothesis was reinforced by the strong predisposition of BMD to hematopoietic cancers such as lymphomas [33,34] and by the fact that a majority of BMDs (58.3% to 66.5%) succumb to cancer, in first place HS, lymphomas, or mast tumors [26,30,35]. “

Often relies on “top SNPs” and their p values, but converting this into understanding by the reader that these SNPs are tagging haplotypes and that haplotype distributions will be useful is not consistent. For example, is the CFA5 locus necessarily the strongest or most influential locus, or are the marker allele frequencies and its haplotype structure in the populations making it more easily discovered by GWA?
Can the authors better provide and explain the haplotype information, numbers and frequencies between cases and controls and how this information resulted in simple regions defined in the figures? Better presenting the complexity of genomic landscape would be very helpful. Better explain the content and results from Tables 2 and 3 in the text. And if there is a limitation to number of Figures/Tables allowed in the main text, shift more of the allotment to the haplotype and loci tables that are now in the Supplement. Please bring out the story around the haplotypes where at most the frequency of a cancer associated haplotype is twice that in the cases than in the controls. Anyway, the discussion of the haplotypes in the text were not enough for me.

As suggested, we have clarified and completed the parts involving haplotypes analysis in the results of HS loci. We now extensively describe their boundaries, frequencies and how they were delimited together with how risk haplotypes were identified. We have better explained the Tables in the main text and we shifted, as suggested, the haplotypes from the Supplementary tables into a new table containing the haplotypes analysis of the CFA 5, 11 and14. As suggested, we insisted on the haplotypes when the frequency of a cancer-associated haplotype is twice that in the cases than in the controls.

I believe this wealth of information can be used to better quantitate the heritability and genetic architecture of HS and perhaps the other 2 cancers as well. The SNP genotype data from the cases and controls could be used to estimate narrow sense heritability (Yang, J., Lee, S.H., Goddard, M.E. and Visscher, P.M. (2011) GCTA: a tool for genome‐wide complex trait analysis. Am. J. Hum. Genet. 88, 76‐82.)

As suggested, we have run the GCTA software to estimate the proportion of phenotypic variance explained by the genotype and added sentences in the results, paragraph 2.3. Lanes 230-236:

”These analyses identified SNVs that were significantly associated with HS risk and were shared between the three predisposed breeds on at least chromosomes 2, 5, 11, and 14. To determine the proportion of HS risk that could be explained by these loci, we performed a restricted maximum likelihood (REML) analysis using GCTA software [40]. All chromosomes together could explain at least 61.8% of the phenotype (*p*-value ≤ 4.93 × 10^−29^; S1 Table ). SNVs of the CFA11 locus could explain over 10.3% (*p*-value ≤ 3.5 × 10^−19^) of the HS phenotype; while, SNVs of the CFA14 locus explained a similar part of the phenotype, and the CFA5 could explain only 4.8–6.7% of the phenotype. ”

Other comments:

Intro on page 6. Transitioning between somatic tissue studies and mutations found there and GWA studies for underlying susceptibility loci in a very long paragraph is not smooth. Perhaps the use of the term “genetic bases” is not specific enough.

We corrected this aspect of the introduction to shorten and smooth this paragraph.

Paragraph 2.1 When stating that the 3 loci are also found / or overlapping in GWA of other cancers can the authors find a way to better demonstrate to us that the regions truly overlap? As it stands they do not give the sequence coordinates routinely and just state a gene name or the chromosome number.
A suggested, we have now added the sequence coordinates and the size of the region delimited by significant SNVs on CFA11. For the CFA20, the updated GWAS allowed the identification of only one significant marker, thus only its position was indicated. In addition we have now included the coordinates of the loci found in previous studies for osteosarcoma, mast cell tumor or lymphomas with the following sentence ;

Lanes 130-132: ” Interestingly, these three regions were previously identified for cancer predisposition in dogs: CFA11 (41.3–41.4 Mb) in osteosarcoma, CFA5 (29.6–34.1 Mb) in lymphomas and hemangiosarcomas, CFA20 (30.9–50.1 Mb) in mast cell tumors [15–17]. »

Paragraphs are missnumbered in Table 1.

We apologize for this mistake which has been corrected in the new version

It is not clear to me how the authors can claim 3 independent peaks on CFA5 for HS and Lymphoma in the combined breeds (Figure 3).

Our claim was based on the fact that Tonomura et al. (2015) ^2^ identified two independent loci located 4 Mb apart on CFA 5, at 29.6 Mb and 34.1 Mb tagging different risk haplotypes and associated with lymphoma and hemangiosarcoma risk in golden retrievers. The GWAS on BMD for HS and lymphoma identified a significant marker on CFA 5, at position 30.5Mb SNPs in high LD with this marker (R2 >0.6) are localized on a region spanning 28.3 Mb to 34.4 Mb. Last, when we combined breeds, the plot of p-values reflects the two independent loci identified by Tonomura et al (2015), But we agreed that we can not conclude that the 3 peaks are independent in the BMD breed and we corrected this with the following sentence:

Lanes 166-169: « These results show that BMDs and golden retrievers share common risk loci on CFA5 that is involved in hematopoietic cancers. Further, the CFA5 locus for both HS and lymphomas in BMD overlaps the two independent loci associated with lymphoma and hemangiosarcoma risk in golden retrievers (29.8 Mb and 33 Mb, respectively; Fig 2). »

.

**Reviewer #2:**

The authors present a Genome-Wide Association Study that evaluates the risk of HS in a cohort of BMDs that was collected through a collaborative network of veterinarians. Samples collected were genotyped using the Illumina 173k and Affymetrix 1.1m SNP arrays that were further imputed for uniformity. GWAS of this data was sequentially complemented with the addition of genotype data from previously collected data for other cancers including Lymphoma and Mast Cell Tumor Cancer. These data sets were used to discover common SNP variants associated to combined cancer risk. Additional GWAS were performed where several breeds were added to refine the analysis. An evaluation of the imputed data was also included along with a sequencing effort to detect functional variants and a CNV assessment of risk of the chr14 loci.

-Overall assessment based on the PLOS GENETICS criteria for publication with a numeric scoring by the reviewer (1-5, five being best):

-Originality (2): The presented paper is very similar to others already published such as Karlsson et al 2013 and Tonomura et al 2015 (cited by the study). It does incorporate some new elements but overall they do not detect highly significant findings that are relevant to the field.

-Importance (2): The reviewer believes that the dog is a very important emerging model for studying cancer. However, this field has been stagnant for some years where discoveries are not followed up. The novel loci discovered in this study a likely going to follow that trend. This is a sad reality of the field.

-Interest to researchers (4): this type of approach is likely to be relevant to researchers in the field and is likely to be cited. Dog papers are very popular.

-Rigor (4): The reviewer believes the data was rigorously evaluated as the methods are quite standard in the field. However, the paper requires further clarification of methodological details and a more through evaluation of the assumptions used (i.e. imputation bias of minor allele frequency SNPs)

-Evidence (2): The study adds a couple candidates to the list of cancer risk genes but does not provide additional evaluation and critique of these new findings. In comparisons to similar papers such as Karlsson and Tonomura's, this paper looks weak

Major concerns:

The paper requires a deep revision to correct grammar and typos. In addition, the paper is written in a very verbose and unnatural language that makes some arguments hard to understand. The reviewer strongly recommends a native English speaker to review and improve the syntax in the paper. There are too many instances to enumerate but some examples are offered at the end.

We apologize for these mistakes. We corrected numerous paragraphs to clarify and improve the syntax of the paper. Moreover for this resubmission, we used the English” Taylor & Francis Editing” language editing service to improve the English of our manuscript.

The variants detected in this study are in large part the same as those already discovered for other cancers (such as osteosarcoma by Karson el al in 2013, cited in the paper). This main concerns is even clearly stated by the authors in section 3.4 "A number of these genes are not only already known to be involved in the predisposition of several cancers (CDKN2A, POT1, FHIT,…) but they are also associated with immune traits (monocyte, platelet…) or with cholesterol, HDL/LDL or allergens traits in Human".

We agree that these loci were already discovered in immune traits or in the predisposition of cancers in Human but this fact reinforced their relevance in canine HS predisposition. The variants detected on CFA11 are close, but different to those detected in osteosarcoma by Karlson el al in 2013 ^3^ (see our comment in the discussion regarding the fact that HS-affected Rottweilers were accumulating risk haplotypes for at least two cancers lanes 419-427). Moreover we not only identified complementary predisposing regions with strong candidate genes (on CFA2, CFA5, CFA14 and CFA20), but we highlighted that these regions are accumulating different risk haplotypes (see modeling approach to evaluate cumulative effects in section 2.5. and below). Finally, to our point of view, it is important to confirm with complementary and independent analyses that CFA 11 locus is an important region in cancers predisposition since the replication study is currently the gold standard for demonstrating that an article is based on solid results. Plos journals support and promote this scientific reproducibility ^4^.

Curiously, this phenomenon is attributed to a pleiotropic effect (see section 3.3); however, also it is discussed in the same section a few lines after, how there are two variants within a 200kb region where one is fixed in the Rottweiler. This argument is counter intuitive because if there are two variants, how can it be pleiotropy?
We agree with the reviewer that pleiotropic loci are traditionally considered as the exact same association signal encompasses multiple cancers (Sud et al. 2017 ^5^) as it was observed in our GWAS in BMD for HS and lymphoma or HS and MCT. But Sud et al. proposed a broader and perhaps more pragmatic definition, that encompasses cancer-specific hot spots with a presumed, shared (but less direct) molecular mechanism. With this definition, for some pleiotropic loci, the immediate cancer-specific mechanism of predisposition may not be shared, though they might ultimately converge on the same oncogenic mechanism. For example, MYC locus harbored risk SNPs for numerous cancers; when they are localized within distinct LD blocks of the MYC locus, they likely reflect tissue-specific effects on cancer risk through the regulation of MYC (Sud et al. 2017)*.* To our point of view, we observe the same situation with the CFA 11 locus in Rottweilers: the Rottweilers cumulate two risk variants: one linked to osteosarcoma risk, fixed in the breed and the other linked to HS risk. To clarify this situation, we modified the title section 3.3 by “*Multi-cancer loci identified through a multi-breed approach”* and we also modified the following sentence:

Lanes 422-427: Co-occurrence of two different risk haplotypes for HS and osteosarcoma across 200 kb also perfectly illustrates that a given locus can harbor multi-cancer risk because of different risk haplotypes as observed in humans. Indeed, in human GWAS, for some loci such as 8q24.21 (containing *MYC*), different risk SNVs are associated with different risk cancers, although they might ultimately converge toward the same oncogenic mechanism [44].

Although the GWAS implicated other novel loci associations, no additional biological argument supported by any other type of technology different from GWAS was provided to support their inclusion. No independent validation cohorts were used.

As suggested, we performed a validation of our findings on two independent cohorts of BMDs by genotyping SNVs from the major loci (CFA5, CFA11, and CFA14). These data clearly strengthened the major role of the risk alleles of these markers in HS development. These results are now presented in a new paragraph: ” *2.7. Validation of the major loci on independent cohorts”,* a new Supplementary Table 4 with the Odds ratios and pvalues and a new author (Caroline Dufaure de Citres), who performed the genotyping of these independent cohorts of BMDs.

It is undisclosed where the samples were collected from. Accounting for samples collected by the study and those added from previously published studies become complicated as they are progressively added into the argument. There are no clear descriptions of the samples in the methods and results that help account for samples included in the study. i.e. The reviewer believes the FCR samples were collected by the researchers but is not completely sure.
We agree that this information was missing from the first version of the manuscript and we have added a description of all samples used in the Method section by the following sentence:

Lanes 528 - 531: «We selected dogs based on their breeds i.e., BMD, Rottweiler, and FCR. For the cases, we included dogs with a pathology report confirming diagnosis of hematopoietic cancer (HS, mast cell tumor, or lymphoma), and for controls, we included dogs >10 years who were without cancer.»

We have also added details regarding the numbers of genotyped dogs using the different arrays in Table 1, as requested by reviewer 3 (see below).

The imputation effort made in the study is concerning even when it has some caveats on his favor. It appears to the reviewer that a 20k genotype dataset was imputed and expanded to a 712k as described in section 4.2. To the reviewer’s opinion, the magnitude of this imputation effort is extreme and very risky. However, to the researcher’s favor, they did this within a breed that are likely to be fixed over large portions of the genome. The reviewer believes it is impossible to determine how appropriate is this approach within the context of dog genomics and the study. In contrast and considering the Minor allele frequency exclusion of 0.01, in humans such threshold would still leave a significant risk of biased SNPs for low frequency SNPs (see Figure 2 in Johnson et al 2013 PMID: 23334152, attached). Although the reviewer considers this section to be plausible, it would be appreciated to have more details to evaluate imputation bias in this specific dog cancer and compared it to a more generalized context.

To answer this important point, also raised by reviewer 3, we have applied a new threshold of 0.05 for filtering the Minor allele frequency (MAF) and have also included the variant Hardy–Weinberg equilibrium (HWE) *p* value >1 × 10^−7^ to consider more stringent parameters. These thresholds were used fo dogs by [Friedenberg](https://www.ncbi.nlm.nih.gov/pubmed/?term=Friedenberg%20SG%5BAuthor%5D&cauthor=true&cauthor_uid=27129452) and [Meurs](https://www.ncbi.nlm.nih.gov/pubmed/?term=Meurs%20KM%5BAuthor%5D&cauthor=true&cauthor_uid=27129452) (2016)^6^. In this latter paper, the authors used the Beagle software to impute 4,885 K SNPs from 84K SNPs (magnitude of imputation of ~x58) with a genotype concordance of 87-92.4%. In our study, the magnitude of imputation is lower (x32 for the 20K to 712K, and x4 for the 170K to the 712K). However, to confirm that our imputation provided reliable results, we used the following strategy: we masked the Affymetrix 712K private SNPs on half of the reference panel and imputed them. The mean concordance between masked autosomal SNVs and imputed SNVs was 91.968% and 95.857% for SNVs imputed from the Illumina 22K to the 712K and for SNVs imputed from the Illumina 173K to 712K respectively. These high concordances reflect the reliability of the imputed genotypes. We corrected the Material and Method section to inform that we used thresholds similar to [Friedenberg](https://www.ncbi.nlm.nih.gov/pubmed/?term=Friedenberg%20SG%5BAuthor%5D&cauthor=true&cauthor_uid=27129452) and [Meurs](https://www.ncbi.nlm.nih.gov/pubmed/?term=Meurs%20KM%5BAuthor%5D&cauthor=true&cauthor_uid=27129452) (2016). Also, we added sentences on the imputation tests confirming the quality of the imputed genotypes at the beginning of the paragraph 2.3 (Lanes 197- 211).

As requested by Reviewer 3, we have added information on our imputation reference panel in the Material and Method. A major weakness comes from the fact that this panel does not contain Rottweilers. Even if BMDs and Rottweilers are phylogenetically closely related, the use of such a reference panel, without a breed, could result in wrong imputed genotypes for Rottweilers. Nevertheless, the shared risk haplotypes identified in these three predisposed breeds contain SNVs from the Illumina 173K SNV Canine HD array and thus only imputed on BMDs genotyped with the 20K (and thus not imputed in the Rottweiler cohort). We added these explanations in the 3.5 paragraph of the discussion, with the following chapter.

Lanes 460-471: Imputation was done to perform GWAS on a larger cohort of cases and controls from the three predisposed breeds with a higher density of SNVs. To avoid incorrect genotypes and potentially unreliable results after imputation, we used parameters previously shown to give accurate imputation in dogs [39]. Both BMD and FCR breeds were included in the reference panel of the higher density SNVs, imputation was performed within the same breeds, and simulations showed a good concordance of the imputed genotypes (91.97–95.86%). However, while there is a close genetic relationship between BMDs and Rottweilers [45,55], the lack of Rottweilers in the reference panel may be a potential limitation. Nevertheless, the shared risk haplotypes identified in the three predisposed breeds had SNVs only from the Illumina 173K SNV Canine HD array; thus, none of them were imputed in the FCR or Rottweiler cohorts (Table 3). Finally, we replicated these results on two independent cohorts of BMDs, and showed the reliability of the loci and risk alleles identified in this study, and their impact on the longevity of BMDs in the whole population.

Even when the sequencing effort was made to detect functional variants, there was no significant findings obtained from it, this is clearly stated in section 3.6. This dramatically limits the impact and relevance of the effort.
We agree that unfortunately the sequencing effort has not allowed the identification of coding variants, this situation is not specific to this study, since like most risk loci of cancer map to non-coding regions in human^5^. Nevertheless it allowed to densify the genotyped variants in the major loci and to point out strong candidates of regulating variants (overlapping in methylation marks or long non-coding RNA).

For the figures, the corrected P-values mentioned in the text do not correspond to what is represented in the figures in the respective vertical axis. i.e. Figure 1 chr5 loci is 6.36E-5 while in the figure is barely above the E-4. This occur in all figures.

This comment may be related to a misunderstanding between logged and unlogged values between the text and the figures. As mentioned in the old Figure 1, the y axis is a –log10 scaled. Because –log10(6.36E-5) = 4.19, the CFA5 locus at 6.36E-5 was barely above the E-4.

For figure 2, besides being a complete chaos of vertical and horizontal mating, the color coding is not legible when printed in a high quality color laser printer. These two issues are likely to make this figure completely useless for the reader.

We acknowledge that pedigree of dogs with numerous (inbred) mattings results in numerous lines. As also requested reviewer 1, we have now moved this Figure in supplementary data.

Some arguments in the paper that are concerning to the reviewer:

In the introduction "and models are strongly needed to better understand this dramatic cancer" --- This a weird statement and an example of the odd writing style used in the paper. What is a dramatic cancer?

We corrected it by “aggressive cancer”.

In the introduction "Furthermore, despite a strong/important heritability of HS in BMD, the awareness of this devastating cancer and attempt to selection against HS since 20 years, breeders have not succeeded to reduce the prevalence of this cancer" --- What evidence does the authors have that suggest there has been any effort to reduce prevalence?

Cancers are responsible of more than half of the death in this breed. HS is particularly devastating in this breed, and clearly impacts the life span of the breed ^7^. Beside the fact that it is a bad publicity for the breed, breeders and clubs have been aware for a long time that if they want to improve the health of the breed they have to select against HS. This cancer was first described in BMD by Moore and Rosin in 1986^8^. Since then clubs and associations of BMD increased the number of health studies and international meetings to inform and fight against this cancer. These efforts resulted in information on HS ^9,10,11,12^ or on available genetic tests ^13,14^, and BMD Clubs set up an International Working Group for BMD health, at which, we and other researchers are regularly invited (since 2006) to inform on HS and cancers in this breed^15,16^. But most important, to fight against this cancer, breeders developed one of the first dog database^17^ to collect health data and samples (BMD Repository Samples) and the AKC Canine Health Foundation has funded at least 13 research grants on this cancer. All these efforts illustrate the willingness of clubs and breeders to improve the health of their dogs by fighting against the major cause of death in their breed.

In section 2.3 "since it is never found in other 231 Swiss dog breeds (18 Appenzeller Sennenhund, 8 Entlebucher Mountain dog and 205 greater Swiss dog, data not shown)..." --- This sentence contains an obvious mistake that makes the sentence very confusing 231 Swiss dog breeds, more like 231 dogs belonging to Swiss breeds. Also, what is the relevance of the Swiss breeds to the BMD and Rottweiler argument when the Rottweiler is not a Swiss breed.
These Swiss breed dogs originate form dogs selected for work in alpine pasture or in the valley of the cantons of Bern, Appenzel, or Entelbuch (these cantons are ~200km far away from the city of Rottweil in Germany). The larger dogs were selected as guard dogs and even if there was not a clearly defined standard, these large dogs were similar. The Bernese mountain dog, the greater Swiss mountain dog and Rottweiler (later, also the St Bernard) all originated from this type ^18^. This close relationship of the Bernese mountain dog, the greater Swiss mountain dog and the Rottweiler is confirmed by recent genetic studies (Fig 4 of ^19^ and Fig 1 of ^20^).

We applogize for the obvious mistake that makes the sentence confusing. To simplify the message, we removed it..

In section 2.5 "When performing the statistical analysis with the imputed genotypes, no coding variant significantly associated with HS risk could be found and six of the ten top variants associated with HS predisposition are imputed genotypes and are localized within 100 kb on CFA11" --- Could this be due to the extreme imputation effort made in the study that is previously described as a concern by the reviewer?
We answered on this imputation effort (see above), but we agree that this could impact the results. We added a sentence in the discussion, section 3.6. to clarify this point:

Lanes 496 – 498: While the genotype concordance of imputed SNVs with the Beagle software was reliable, imputation of SNVs identified by the capture could limit the detection of causal variants.

In section 3.2 " the cumulative risk alleles on the 3 main loci (CFA11, 5, 14) strongly impact the probability to develop this cancer with an Odd ratio of 5.41 to be affected by HS when dogs carry 5/6 risk alleles" --- This is an important finding to the reviewer that is not described or discussed.

This comment is related to reviewer 1’s comment. As suggested we have conducted a more extensive characterization of the haplotypes analyses, in result sections 2.4 and 2.5 and have included the previous supplementary table to the main text (now Table 4).

In section 3.6 "With multiple breeds and cancers approach, we highlight the cumulative effect of different risk haplotypes behind each locus and their pleiotropic nature" --- the reviewer is of the opinion that to make this statement it would have been necessary a modeling approach such as the one presented by Zapata et al 2019 where cumulative effects are expressly evaluated and collinearity is considered.

First, the fact that cumulative 5/6 risk alleles strongly impact the probability to develop HS illustrates this cumulative effect. Nevertheless, as suggested by the reviewer, we performed 2 Stepwise model selections (a Stepwise Forward selection model inspired from Zapata et al. 2019 and a Stepwise both Forward and Backward model selection) on all 1,505 significant SNVs from the GWAS 8 to select the best predictive models. The best models include multiple markers of CFA2, 5, 11 and 14 showing the cumulative effect of different risk haplotypes. We have added these data to the Result part, and also to a new chapter 4.4., in the Material and Methods section “Predictive modeling”: Lanes 592 - 605.

Finally, we notice that the Reviewer 2 point out that if the genotype data are available in the research team Web page, it is possible to track people who will access to the data. The release of these GWAS data on each Institute website is a common practice; indeed GWAS data from ^2,3, 21^ are available on the Broad Institute’s website. We propose to submit the complete data set of genotypes and phenotypes to the Dryad Repository

**Reviewer #3: Comments to the author:**

This study aims to identify loci associated with histiocytic sarcoma in the three dog breeds most at-risk for this cancer. To increase GWAS statistical power, the authors:
a) increase sample size by including phenotypes for several closely related cancers (like lymphoma and mast cell tumor), making use of previously published data,
b) increase sample size by combining cases and controls from several breeds known to have a predisposed risk (like golden retriever and Rottweiler)
c) increase the number of SNPs to test by using several different arrays (ranging from a 22k custom capture array to the 1.1M Affymetrix array) and imputing between these.

Significantly associated loci on 8 different chromosomes are identified – some also found in previous studies – and the authors follow up on several of these by investigating haplotypes, using targeted sequence capture and performing methylation analyses. The authors find several risk (or protective) haplotypes that are mostly shared between breeds and some even between the different cancer types, and which are regulatory in nature. This is a thorough and interesting research study, which propels the field of canine complex disease genetics.

The manuscript is generally written well and easy to follow. But there are many minor English grammatical errors.

We appreciate the Reviewer’s enthusiasm and evaluation our study

Main comments:

1. Phenotyping – no details at all are provided on how cases and controls were selected. What criteria were used (apart from breed)? Was there a minimum age for controls?
We agree that this information was missing from the first version of the manuscript and we have added a description of all samples used in the Method section by the following sentence:

Lanes 528 – 531: We selected dogs based on their breeds i.e., BMD, Rottweiler, and FCR. For the cases, we included dogs with a pathology report confirming diagnosis of hematopoietic cancer (HS, mast cell tumor, or lymphoma), and for controls, we included dogs >10 years who were without cancer

2. Quality control – what other filters did you use on your genotyping data? Only MAF of 1% is mentioned. What about SNVs that failed Hardy-Weinberg? Or SNVs with high missingness? Did you do a sex check and a breed check (like a PCA) to make sure there were no sample mixups? What was the final SNV number left for analysis after these filters? More details are required.

We agree on the lack of details for the filtering part. For GWAS, we did not perform a filter with Hardy-Weinberg since many genomic regions of dogs are not following Hardy-Weinberg equilibrium. But for the imputation, to respect Friedenberg and Meurs ^6^ criteria (see above), we only imputed the SNVs respecting HWE *p*value >1×10^−7^ and MAF 0.05. For GWAS, SNVs genotypes were filtered for per sample call rate >95%, per SNV call rate >95% and MAF 0.01. We have checked the breeds thanks to a cluster tree illustrating genetic distances between individuals (Cf Figure a the end of this document). Checking for the sex status was performed thanks to the Plink option “--check-sex “. We clarified these points in the Material and Method section. Moreover, we have now added the number of remaining SNVs in the different GWAS sections.

3. Permutations – I’m a little concerned that the thresholds were all set based on only 500 permutations. 10,000 is a much more reasonable and usual number, but at least 1,000 permutations should be used. Also, more details about how these permutations were run is needed – program used, etc. And how was the threshold actually calculated? Some of the “significant” P-values are very low…certainly much lower than the Bonferroni correction (although realizing this is overly conservative).

As requested we re-run the analysis and performed 1000 permutations with Eigenstrat – GenABEL 1.8. We identified all SNVs with either significant association (exceeding 95% confidence intervals defined empirically using 1,000 random phenotype permutations with Eigenstrat – GenABEL 1.8) This method used by Karlsson et al.^3^ is less conservative than Bonferroni correction. We clarifed the details of permutation in the Material and Methods section, Lanes 544 – 546.

4. There are no imputation accuracy metrics given or tests run. Or even previous papers showing canine imputation accuracy cited. How do we know the imputation worked well and provides reliable results?
This point was also raised by Reviewer 2 and we corrected it (see above)

Minor comments: (Note: there are no page numbers or line numbers so that makes this difficult)
1. I’m not a fan of the first part of the title

We removed it

2. Write out the gene names the first time they are used

We corrected it.

3. Introduction, 2nd paragraph – “clinical presentation, histopathology and issue of this canine cancer…” – not sure what is meant by “issue”, remove.

We removed it (meant outcome).

4. Introduction, 2nd paragraph – “attempt to selection against HS since 20 years, breeders have not succeeded to reduce the prevalence” should be “attempt to select against HS for 20 years, breeders have not succeeded in reducing the prevalence”

We corrected it.

5. Introduction, 3rd paragraph – “showed that they are also associated to other cancers in predisposed breeds” should be “with other cancers”

We corrected it.

6. I think fig. 2 should be supplemental – it doesn’t show your results and is more like a side-note

We moved it as a supplemental figure.

7. Fig. 4 – addition of 44 cases with MCT to the HS data. But the number of controls decreases from 154 to 135 – why?

This is a good remark. The risk to develop a cancer increases with age and some old dogs (>10 year-old) developed at old age MCT or lymphoma, which are common cancers in dogs. These dogs were used as controls for HS but as cases for MCT or lymphomas. This explained why the control number decreased. However, in this revised version, we removed these controls from the GWAS and re-run all analyses.

8. Section 2.3 last paragraph – “specific to BMD and Rottweilers HS cases since it is never found in other 231 Swiss dog breeds” should be “specific to BMD and Rottweiler HS cases since it is never found in a further 231 dogs of other Swiss breeds”

The sentence has been deleted following one comment of reviewer 2.

9. Section 2.3 last paragraph, last line – should be “close” not “closed”

We corrected it at other occurrences.

10. Fig 5 & 6 - remove question marks in boxes next to A, B, C, etc

They were absent in submitted Figures; we will be vigilant to ensure that the pdf converted version do not contains these question marks.

11. Section 2.4, 1st paragraph – 134 dogs were genotyped on Affymetrix array – more detail about the dogs selected – what breeds? Add to methods.

We added this information in the methods.

12. Section 2.4, 4th paragraph “no common risk haplotype can been identified with higher SNV…” should be “can be identified”

This sentence was removed.

13. Section 2.4, last three words should be “Supplementary Table 5”.

We corrected it.

14. Section 2.5, 1st paragraph – §3.4 doesn’t seem relevant to this sentence.

We corrected it.

15. Section 2.5 – either use commas or spaces to separate the millions and thousands in bp coordinates but not both

We corrected it at other occurrences

16. Section 2.5 – the top ten variants are shown but how many significant SNVs were actually identified for each of these GWAS?

We corrected it.

17. Section 2.5, 2nd paragraph – where is the data from the GWAS using the CFA11 best SNV as a covariate? Add the top ten results to Supp. Table 6.

We mentioned it in the new Supp.table 3.

18. Discussion – small “h” on human

We corrected all occurences

19. Discussion, 3.1, 2nd sentence – switch the words “common” and “loci”

We corrected it

20. Discussion, 3.1, 3rd sentence needs rewriting. Maybe: “Interestingly, somatic alterations identified to date in canine tumors, through genome-wide approaches, are found in the same genes”

We corrected it

21. Discussion, 3.2 title – remove the “of” after cumulative

We corrected it

22. Discussion, 3.2, 2nd sentence needs rewriting. Maybe: “Within-breed canine GWAS usually identifies fewer variants with stronger…”

We corrected it

23. Discussion, 3.2, 3rd sentence – replace “beauty” with “morphological”

We corrected it

24. Discussion, 3.2, middle of the paragraph – need a fullstop after “Rottweilers” and then on the same line remove the repeated words “a strong”

We corrected it

25. Discussion, 3.2, last line – add in years for Arendt et al and Tonomura et al

We included the dates.

26. Discussion, 3.4 – please put P-values into scientific notation and remove the … in the lists in brackets

We corrected it

27. Discussion, 3.4, last line – start needs rewriting. Maybe: “Concomitantly to this work, a study by Labadie et al confirmed the pleiotropic effect of these canine cancer loci by identifying a shared region for canine T zone lymphoma,…”

We corrected it

28. Discussion, 3.5, 2nd sentence – use “The CFA5 locus…” 3rd sentence – use “Very little is known…”

We corrected both.

29. Discussion, 3.5, 1st paragraph, reference needed for sentence ending in “associated in human GWAS with the chemokine CCL2.”

We added Ref 57 (Ramos et al., 2014)

30. Discussion, 3.5, 2nd paragraph – use “difference in age” instead of “difference of the age”

We corrected it

31. Discussion, 3.5, 3rd paragraph – use “The CFA20 locus..” Reference needed for sentence ending in “involved in human predisposition of breast cancer.”

We corrected it and we added Ref 57 (Ramos et al., 2014)

32. Supplementary figure 5 caption – change all occurrences of “copy” to “copies” after either zero or two, i.e., should be “zero copies” and “two copies”. Also, second occurrence of “Cases with one copy” should be “Cases with two copies” and second occurrence of “Controls with one copy” should be “Controls with two copies”.

We apologize for these mistakes and corrected them.

33. Methods – give the full name for abbreviations, like Cani-DNA BRC, CNRS, CNG

We corrected it.

34. Methods, 4.1 – change the Ulve et al citation to the corresponding reference number

We corrected it.

35. Methods, 4.1, 3rd sentence – should be “veterinarians” and no s on “medical care”

We corrected it.

36. Methods, 4.2 – what numbers of dogs from what breeds were genotyped on the different arrays? Details missing.

We added the details in Table 1 and referred to it in the Methods.

37. Methods, 4.2 – GenAbel 1.8 – is this an R package? If so, you should state this.

We corrected it.

38. Methods, 4.3, 3rd paragraph – small q on qPCR

We corrected it.

39. Methods, 4.3, last sentence should read: “It consists of gene annotations (RefSeq), and detection…”

We corrected it.

40. Table 1 – paragraph numbers are wrong

We corrected it.

41. Supplementary table 6 – please explain the last column, Pc1df

We corrected it by Pcorrected.

42. Supplementary table 7 – it would help if you added the chromosomes (and approx. bp) for these genes

We corrected it.

**References**:

1.<https://www.bmdca.org/health/pdf/2005_Health_Survey.pdf>

2. Tonomura N, Elvers I, Thomas R, Megquier K, Turner-Maier J, Howald C, et al. Genome-wide association study identifies shared risk loci common to two malignancies in golden retrievers. PLoS Genet. 2015;11: e1004922. doi:10.1371/journal.pgen.1004922

3. Karlsson EK, Sigurdsson S, Ivansson E, Thomas R, Elvers I, Wright J, et al. Genome-wide analyses implicate 33 loci in heritable dog osteosarcoma, including regulatory variants near CDKN2A/B. Genome Biol. 2013;14. doi:10.1186/gb-2013-14-12-r132

4. <https://theplosblog.plos.org/2020/04/the-importance-of-being-second-plos-wide-edition>

5. Sud A, Kinnersley B, Houlston RS. Genome-wide association studies of cancer: current insights and future perspectives. Nat Rev Cancer. 2017;17: 692–704. doi:10.1038/nrc.2017.82

6. Friedenberg SG, Meurs KM. Genotype imputation in the domestic dog. Mamm Genome Off J Int Mamm Genome Soc. 2016;27: 485–494. doi:10.1007/s00335-016-9636-9

7. Klopfenstein M, Howard J, Rossetti M, Geissbühler U. Life expectancy and causes of death in Bernese mountain dogs in Switzerland. BMC Veterinary Research 2016 July 25, 12 (1): 153

8. Moore PF, Rosin A: Malignant histiocytosis of Bernese mountain dogs. Vet Pathol 23:1–10, 1986

9. <https://www.bmdca.org/health/studies.php>

10. <https://www.bmdca.org/health/pdf/2000_Health_Survey.pdf>

11. <https://www.bmdca.org/health/pdf/2005_Health_Survey.pdf>

12. <http://www.bmdinfo.org/Health/Histiocytic_Diseases_of_the_Bernese_Mountain_Dog.php>

13. <http://www.bmdca.org/health/tests.php>

14. <http://berner-iwg.org/index.php/resources/>

15. http://berner-iwg.org/index.php/2006-como-italy/

16. <http://berner-iwg.org/index.php/2018-amsterdam-the-netherlands/>

17. <http://bernergarde.org/db/>

18. Book : The bernese mountain Dog –Yesterday and Today_from Silvana Vogel Tedeschi p15. Edizioni Al Trovante.

19. Ostrander EA, Wang G-D, Larson G, vonHoldt BM, Davis BW, Jagannathan V, et al. Dog10K: an international sequencing effort to advance studies of canine domestication, phenotypes and health. Natl Sci Rev. 2019;6: 810–824. doi:10.1093/nsr/nwz049

20. Vaysse A, Ratnakumar A, Derrien T, Axelsson E, Rosengren Pielberg G, Sigurdsson S, et al. Identification of genomic regions associated with phenotypic variation between dog breeds using selection mapping. PLoS Genet. 2011;7: e1002316. doi:10.1371/journal.pgen.1002316

21. Arendt ML, Melin M, Tonomura N, Koltookian M, Courtay-Cahen C, Flindall N, et al. Genome-Wide Association Study of Golden Retrievers Identifies Germ-Line Risk Factors Predisposing to Mast Cell Tumours. PLoS Genet. 2015;11: e1005647. doi:10.1371/journal.pgen.1005647

Figure: Cluster tree illustrating the genetic distances between individuals genotyped in this study and publically available ^2,3,21^. This cluster tree shows that there are no sample mixups between breeds.
